# Triaxial Creep Characteristics and Constitutive Model of Argillaceous Siltstone Under Water-Induced Deterioration in the Lower Minjiang River

**DOI:** 10.3390/ma19214637

**Published:** 2026-10-31

**Authors:** Bo He, Li Zhuo, Yu-Hao Wang, Zheng Chen, Huai-Zhong Liu, Ming-Li Xiao, Hong-Qiang Xie

**Affiliations:** 1 Sichuan Minjiang Port & Shipping & Electricity Power Development Co., Ltd., Leshan 614000, China; hb14678@163.com (B.H.); wangyuhao19960820@163.com (Y.-H.W.); cz15284801711@outlook.com (Z.C.); 2 State Key Laboratory of Hydraulics and Mountain River Engineering, College of Water Resource and Hydropower, Sichuan University, Chengdu 610065, China; huaizhong.liu@scu.edu.cn (H.-Z.L.); xiaomingli@scu.edu.cn (M.-L.X.); alex_xhq@scu.edu.cn (H.-Q.X.)

**Keywords:** argillaceous siltstone, water-induced deterioration, triaxial creep test, long-term strength, creep constitutive model

## Abstract

To investigate the time-dependent deformation behavior of argillaceous siltstone under water-induced deterioration and confining pressure, step-loading triaxial creep tests were conducted on specimens from the lower Minjiang River under air-dried and saturated conditions at confining pressures of 4, 6, and 8 MPa. The effects of water-wetting on creep deformation, the steady-state creep rate, and long-term strength were analyzed. A three-dimensional nonlinear viscoelastic–plastic creep model was developed by introducing a moisture–stress coupled damage factor and a nonlinear viscoplastic component into the Burgers model. The results show that water-wetting leads to a substantial reduction in long-term strength by 28.4–37.5% and increases the steady-state creep rate. Increasing the confining pressure enhances the long-term bearing capacity, with the failure axial stress rising from 56.02 MPa to 81.09 MPa for air-dried specimens and from 37.10 MPa to 56.38 MPa for saturated specimens. The reduction in shear strength parameters is mainly reflected in cohesion decreasing from 6.72 MPa to 2.66 MPa, while the internal friction angle changes minimally. The proposed model effectively captures the full creep process under various stress levels, with the coefficients of determination mostly exceeding 0.90, demonstrating its applicability for describing the triaxial creep behavior of siltstone in the study area.

## 1. Introduction

The creep constitutive model serves as an important bridge connecting experimental phenomena with engineering analysis and also provides the theoretical basis for predicting the long-term stability of soft rocks [1,2]. For red-bed siltstone in a basin-margin region, its mechanical response is simultaneously controlled by multiple factors, including the stress level, water-wetting environment, structural damage, and time effects. Red beds are continental clastic sedimentary sequences dominated by reddish sandstones and mudstones, typically deposited in arid or semi-arid environments, with their red color mainly due to iron oxide (hematite) coatings on the grains. It is difficult to realistically model the long-term evolution of red-bed deformation solely based on instantaneous strength parameters or empirical reduction coefficients [3,4]. Therefore, scholars worldwide have carried out extensive research on the creep constitutive relationship of red-bed siltstone, with research approaches gradually evolving from classical empirical and combined element models to improved models that consider damage evolution, water-wetting coupling, and nonlinear deformation characteristics [5,6,7,8].

In terms of classical models, the Maxwell, Kelvin, Burgers, and Nishihara models, due to their relatively simple forms and clearly defined physical meanings of parameters, have been widely used to describe decelerating creep, steady-state creep, and viscoelastic–plastic deformation in soft rocks [9,10,11]. Yang et al. [12] proposed an analytical method for back-analysis of rock mass compressive creep parameters based on field plate loading tests. Wei et al. [13] further developed a unified framework for creep models and parameter inversion by combining in situ double-propped plate loading tests on columnar jointed basalt. Gan et al. [14] introduced a nonlinear finite element method into the back-analysis of creep parameters. These studies all demonstrate the strong practicality of classical models in interpreting in situ test data and back-analyzing engineering parameters. For typical red-bed mudstone, Deng et al. [15] revealed the creep deterioration mechanism of red-bed soft rock through water–rock interaction tests. Studies by Miao et al. [16] and Sun et al. [17] showed that the Burgers-type model and its modified forms can reasonably reflect the basic characteristics of the decelerating creep and steady-state creep stages of mudstone under different water contents and confining pressures, making them one of the most widely applied basic model forms in current engineering analysis.

However, traditional combined component models still exhibit significant limitations when describing the complex rheological behavior of mudstone. First, classical models are mostly based on linear viscoelastic or ideal viscoplastic assumptions, making it difficult to adequately characterize the nonlinear deformation and damage accumulation effects exhibited by materials under medium-to-high stress conditions [18,19]. Second, for structurally sensitive rocks such as mudstone, although classical models can fit certain stages of the creep curves reasonably well, they lack sufficient capability to describe the entire process from decelerating creep and steady-state creep to accelerating creep and are particularly inadequate when representing structural deterioration and the progressive degradation of long-term strength [20,21]. Liu et al. [1] and Cao et al. [5] pointed out that soft rock under high stress exhibits significant nonlinear characteristics during the accelerating creep stage, and traditional linear combined element models struggle to accurately capture the deformation trend at this stage. Third, in situ creep data often exhibit scale effects, environmental dependence, and staged deformation characteristics. It is difficult for models based solely on linear combined elements to simultaneously determine parameter stability, physical interpretability, and engineering applicability [22,23]. Therefore, introducing damage variables, water-wetting coupling terms, and nonlinear viscous components into traditional models has become an inevitable trend for improving model description capability.

In recent years, nonlinear creep models considering time-dependent damage and environmental effects have become a research hotspot. Zhang et al. [24] established a creep damage model incorporating a freeze–thaw damage factor through creep tests on white sandstone under short-term freeze–thaw cycles. Deng et al. [25], Zhang et al. [26], and Wu et al. [27] conducted research focusing on water–rock interaction, hydro-mechanical coupled damage, and hydrochemical environmental influences, highlighting that water-bearing conditions not only affect the creep rate but also alter the time-dependent evolution law of model parameters. Mudstone in water-rich environments often exhibits pronounced structural deterioration, parameter degradation, and rheological acceleration. Zheng et al. [28] investigated the creep characteristics of water-bearing soft rock through triaxial unloading creep tests and proposed a nonlinear elasto-viscoplastic creep model incorporating a time-dependent water–stress coupled damage function. Li et al. [29] revealed the controlling effect of initial damage state on creep evolution based on creep mechanical property tests of water-bearing rock-like materials at different damage levels, providing an experimental basis for constructing damage-coupled models. Li et al. [4], Luo et al. [30], and Chen et al. [31] highlighted that acidic environments significantly affect the long-term strength and accelerated failure process of weak interlayers, indicating that creep models for interlayers also need to account for environmental deterioration effects. Furthermore, improved models based on damage theory and nonlinear viscous elements are more suitable for complex soft rock materials. Zheng et al. [32] studied the effects of water-bearing deterioration and overstress on concrete creep from the perspective of damage rheology and established a creep model coupling water-bearing deterioration and stress damage. Peng et al. [33] and Hu et al. [34] developed nonlinear creep models from the perspectives of hydro-mechanical coupling and mudstone strength parameter damage, respectively, significantly improving descriptions of the accelerating creep stage and long-term stable state. Wang et al. [35] considered the damaging effect of dry–wet cycles on reservoir bank slope rock masses and established a corresponding nonlinear creep damage model. Chen [36] combined the Nishihara model with an elasto-plastic damage model based on the mesoscopic heterogeneity of sandstone and established a creep damage model capable of reflecting microcrack propagation. Liang et al. [37] discovered the transformation trend of stiffness, first strengthening and then weakening, during rock deformation through cyclic loading–unloading creep tests, and proposed a rheological model containing the strengthening–weakening effects. Based on this, further studies focusing on crack propagation damage mechanisms, fractional-order theory, and specialized models for weak interlayers have expanded the nonlinear modeling approach [38,39,40,41,42,43].

In summary, existing research has developed from classical combined component models toward nonlinear, damage-based, and environment-coupled models. For red-bed siltstone in the margin region of the Sichuan basin, there remains a need to further establish creep constitutive models that can better characterize water-wetting effects, time-dependent damage, and long-term deformation evolution. To this end, this study takes argillaceous siltstone from the dam site of a hydropower project in the lower Minjiang River as the research object; conducts step-loading triaxial compression creep tests; and investigates the creep deformation characteristics and long-term strength of argillaceous siltstone under different water-bearing states. Furthermore, a nonlinear viscoplastic element is introduced into the Burgers model to construct a nonlinear viscoelastic–plastic creep constitutive model considering moisture–stress coupled time-dependent damage. The Levenberg–Marquardt nonlinear regression method is employed for model parameter identification, providing theoretical support for the long-term stability evaluation of red-bed siltstone dam foundation rock masses in the riverbed.

## 2. Triaxial Compression Creep Testing of Argillaceous Siltstone

### 2.1. Mineral Composition and Microstructure of Argillaceous Siltstone

**Figure 1:**
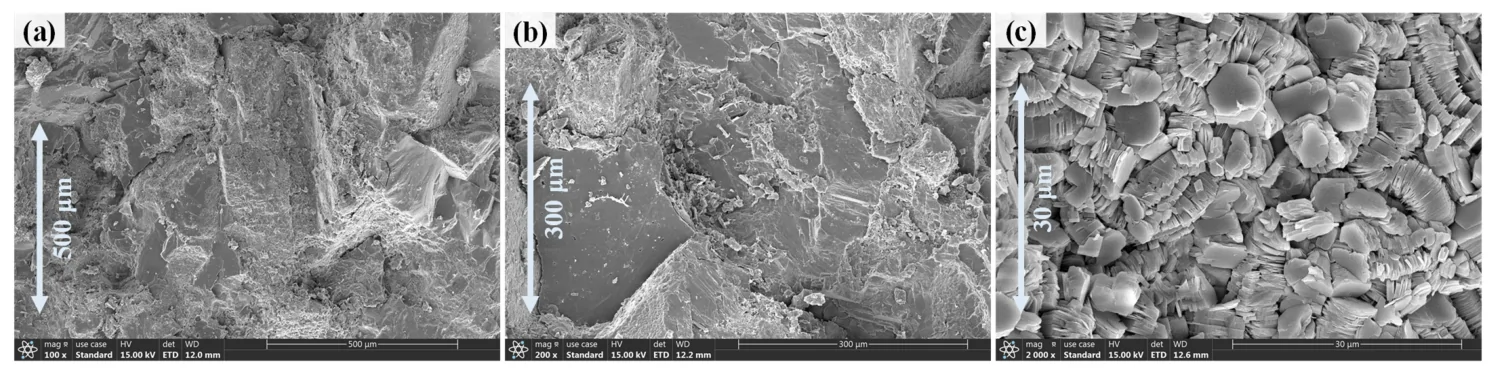
Electron microscope images of argillaceous siltstone microstructure magnified (**a**) 100 times, (**b**) 200 times, and (**c**) 2000 times.

The rock samples used in this test were all taken from the dam site of a hydropower project in the lower Minjiang River, with the lithology being mainly argillaceous siltstone. The X-ray diffraction (XRD) test was used to quantify the following mineral abundances of the main crystalline phases: quartz (40%), kaolinite (20%), feldspar (10%), and calcite (7%), with the remaining 23% corresponding to the minor phases chlorite, muscovite, and other minerals. The mineral phases were identified by matching the measured diffraction peaks to the standard powder diffraction data. The contents of the main crystalline phases were quantified using the reference intensity ratio method. Figure 1 shows the electron microscope images of the mesoscopic mineral particle structure of the rock mass, showing that the argillaceous siltstone is dominated by a clastic particle structure, with particles mostly sub-angular in shape, arranged relatively densely, and surrounded by cement. Specifically, the lower-magnification images show the detrital grain framework, intergranular pores, and cementation between particles; the highest-magnification image clearly reveals platy clay minerals filling the intergranular pores. The overall structure is relatively intact, indicating that the argillaceous siltstone has strong particle support and cementation and relatively good mechanical properties overall.

### 2.2. Specimen Preparation

To ensure that the test results have low discreteness and are representative, large rock blocks with good integrity and no obvious macroscopic fractures were first selected on site, sealed and wrapped, and then transported to the indoor laboratory. Cores were drilled using a coring machine and, using a rock cutter and a double-sided grinder, standard cylindrical specimens with a diameter of 50 mm and a height of 100 mm were produced. During processing, specimen accuracy was strictly controlled to ensure that the parallelism error of the two end faces was less than 0.05 mm and the perpendicularity deviation of the end faces was less than 0.25°.

Micro-fractures and heterogeneity inside natural rock may significantly interfere with the test results, so acoustic wave tests were conducted on all specimens after specimen processing. Specimens with abnormal wave velocity and density were excluded, and those with similar longitudinal wave velocities were selected as formal test objects to ensure consistency between the initial physical and mechanical properties of the specimens. These specimens were then divided into an air-dried group and a saturated group. The air-dried specimens were stored in a ventilated room at around 20 °C and 50–60% relative humidity for at least 30 days until mass stabilized. The saturated specimens were wrapped with gauze and PVC mesh, placed in a humidity box (>95% relative humidity) at room temperature, and weighed daily until the mass change was <0.1 g over 48 h. The water-bearing state, wave velocity, mass, and water content of each specimen are shown in Table 1. Due to the long duration of each test, only one specimen was tested per condition; therefore, the observed differences may partly reflect specimen-to-specimen variability. Despite careful selection, some variation in longitudinal wave velocity remained among the specimens (1307–1922 m/s), reflecting the inherent heterogeneity of the natural rock material. Such differences in initial microfabric and density may influence the magnitude of instantaneous strain and steady-state creep rate under identical loading conditions. However, the overall trends—namely, the reduction in long-term strength, increase in creep strain upon saturation, and the suppression of creep deformation with increasing confining pressure—are consistently observed across all specimens. Therefore, while specimen heterogeneity introduces quantitative scatter, it does not alter the qualitative conclusions drawn from the comparative analysis of water-bearing states and confining pressures.

### 2.3. Test Apparatus and Scheme

**Figure 2:**
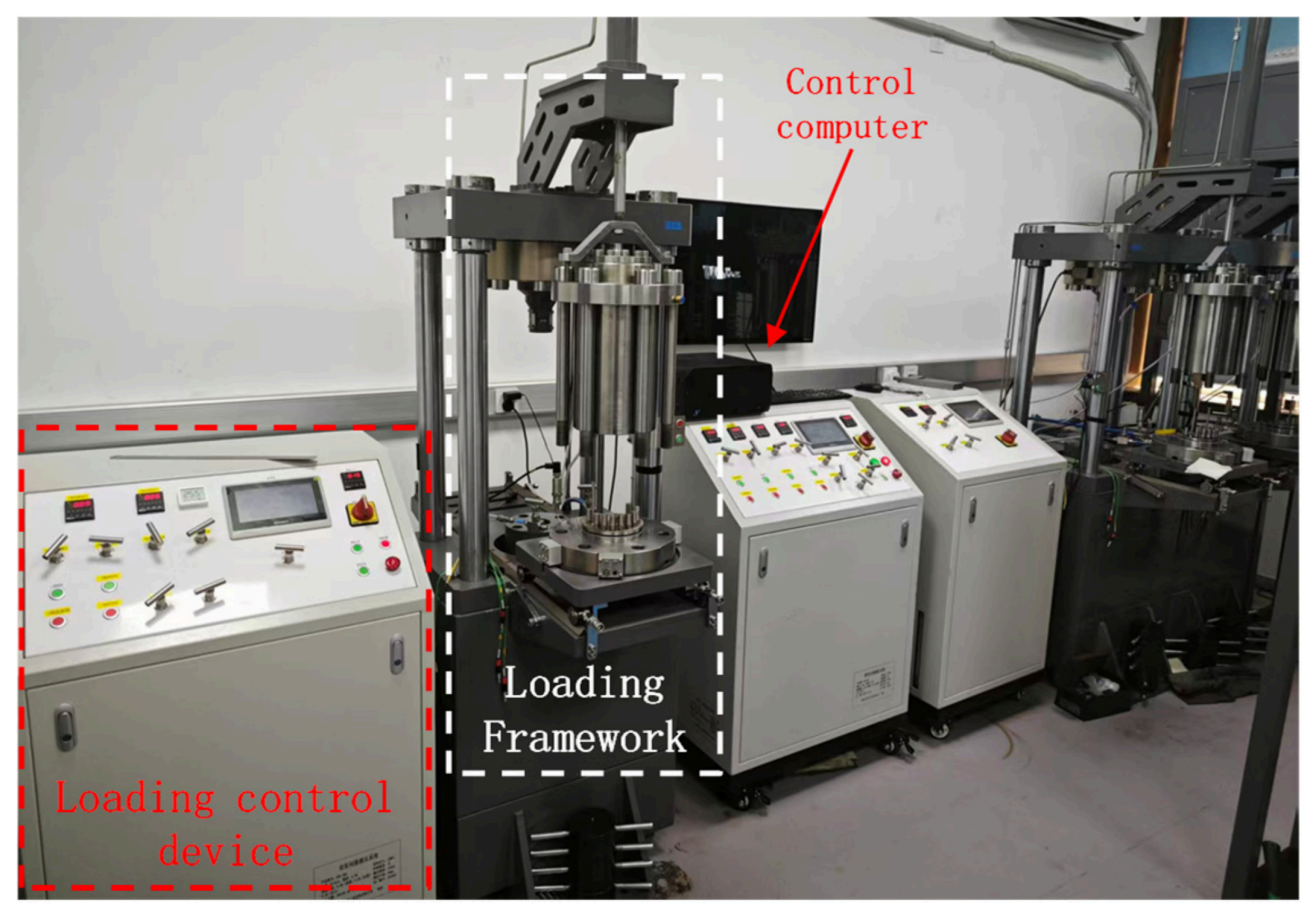
BRJX-100 multi-field coupled rock triaxial creep test system.

In this study, a triaxial creep test was completed on the self-developed BRJX-100 multi-field coupled rock triaxial creep test system, as shown in Figure 2. This system mainly consists of a seepage field control unit, a triaxial stress loading and control unit, and a data acquisition unit. The stress loading and control unit precisely servo controls the axial pressure and confining pressure during the test through an electric-driven servo pump. These control units work independently of each other and cooperate with the multi-channel data acquisition unit to execute long-term creep tests of specimens under independent stress paths.

All creep tests were carried out under undrained conditions, which were achieved by completely sealing the specimen as follows: The upper and lower loading platens were both impermeable and non-drainable, providing no drainage path at either end of the specimen, while the circumferential surface of the specimen was tightly enclosed by a membrane, preventing any fluid exchange or drainage through the lateral surface. Since both the end boundaries and the lateral jacket were impermeable, no fluid was allowed to enter or leave the specimen during the entire creep process. This sealing method also maintained the prepared water-bearing state (air-dried or saturated) of each specimen during the long-term test. The tests were performed at a room temperature of 26 ± 1 °C, maintained by the laboratory air-conditioning system. Axial deformation was measured using linear variable differential transformer (LVDT) displacement transducers with a resolution of ±1 μm and a thermal drift coefficient of 0.02%/°C. Because the temperature variation during testing was within ±1 °C (i.e., a total span of 2 °C), the maximum influence of thermal drift on the measured deformation is less than 0.04%. Therefore, the thermal drift of the transducers is negligible and does not affect the measured creep deformation or the reported creep strain rates.

**Figure 3:**
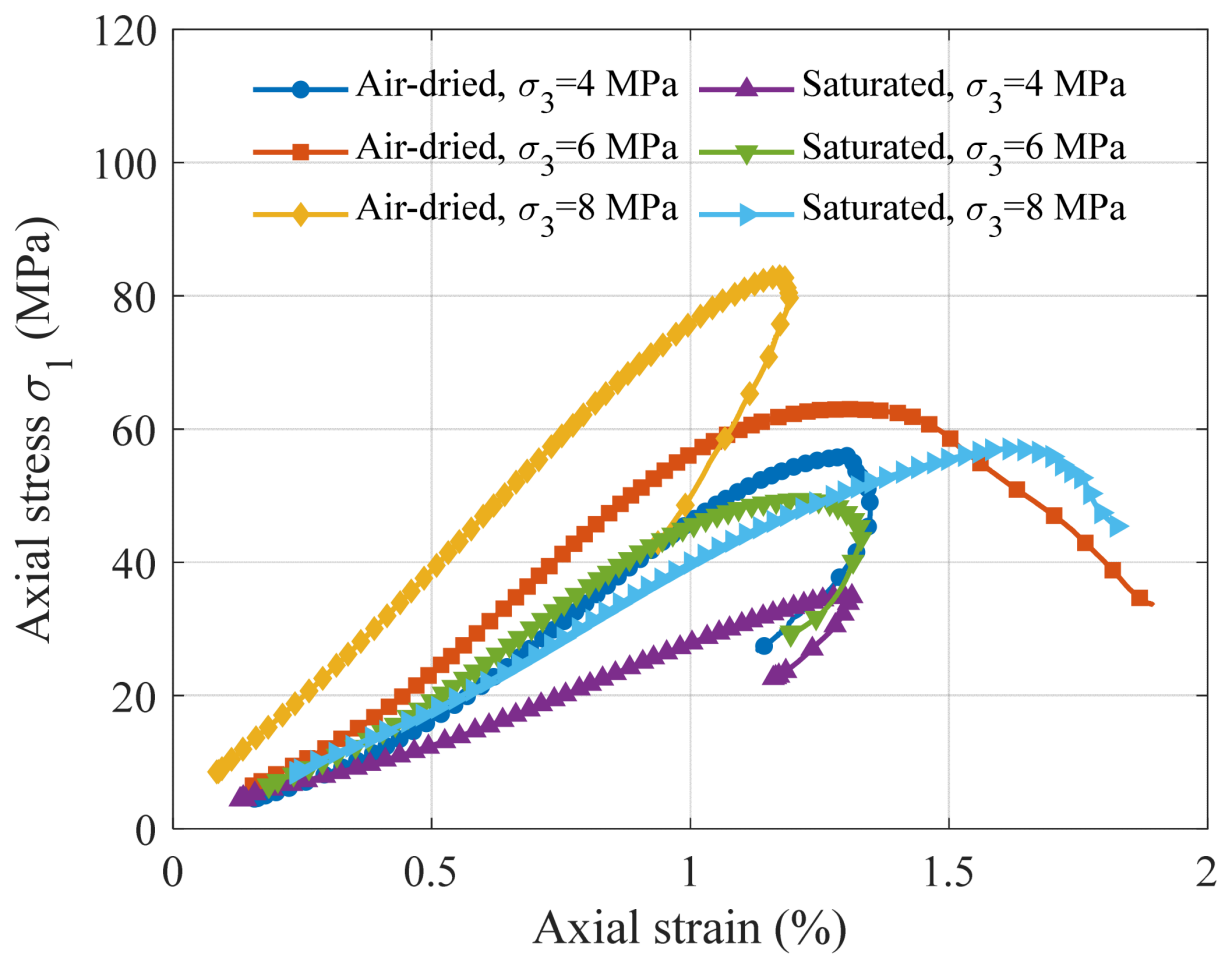
Conventional triaxial stress–strain curve.

To systematically reveal the time-dependent deformation characteristics of argillaceous siltstone under the coupled effect of water-induced deterioration and confining pressure, a detailed triaxial compression creep test scheme was devised based on the strength characteristics of argillaceous siltstone. Three typical confining pressures of 4 MPa, 6 MPa, and 8 MPa, representing different in situ stress levels, were selected. To address the core research objective—determining the influence of water content on the creep mechanical properties of rock foundations—two different water-bearing states, air-dried and saturated, were selected to study the mechanical properties of the rock throughout the process from natural air-drying to complete saturation. The test loading adopted a step-loading mode with constant confining pressure. The axial stress was applied stepwise according to a preset fixed increment, with a preset number of loading steps of 4 to 6. The creep duration under each load level was fixed at a minimum of 96 h. If, after 96 h, the axial strain rate had not yet stabilized, the load was maintained for a longer period until a steady state was observed; only then was the axial stress increased to the next level. This procedure was repeated until the specimen finally underwent accelerating creep failure. According to the above principles, the specific working condition combinations shown in Table 2 were determined for comparative tests. Prior to the creep tests, conventional triaxial compression tests were carried out on the same batch of argillaceous siltstone to determine the short-term peak strength, which provided the basis for designing the multi-level step-loading scheme. One conventional test was performed per condition. The tests were conducted under displacement control at a loading rate of 0.06 mm/min, and the axial stress–axial strain curves obtained are presented in Figure 3. The short-term peak strengths under confining pressures of 4, 6, and 8 MPa were approximately 55.99, 63.00, and 82.89 MPa for air-dried specimens, and approximately 35.06, 49.38, and 57.02 MPa for saturated specimens, respectively. The first few load levels of the creep tests were set below the corresponding short-term peak strength, and the final load level was close to it, so that each specimen experienced decelerating and steady creep first and then experienced accelerating creep.

## 3. Triaxial Creep Mechanical Properties of Argillaceous Siltstone Under Water-Induced Deterioration

### 3.1. Creep Deformation Characteristics

**Figure 4:**
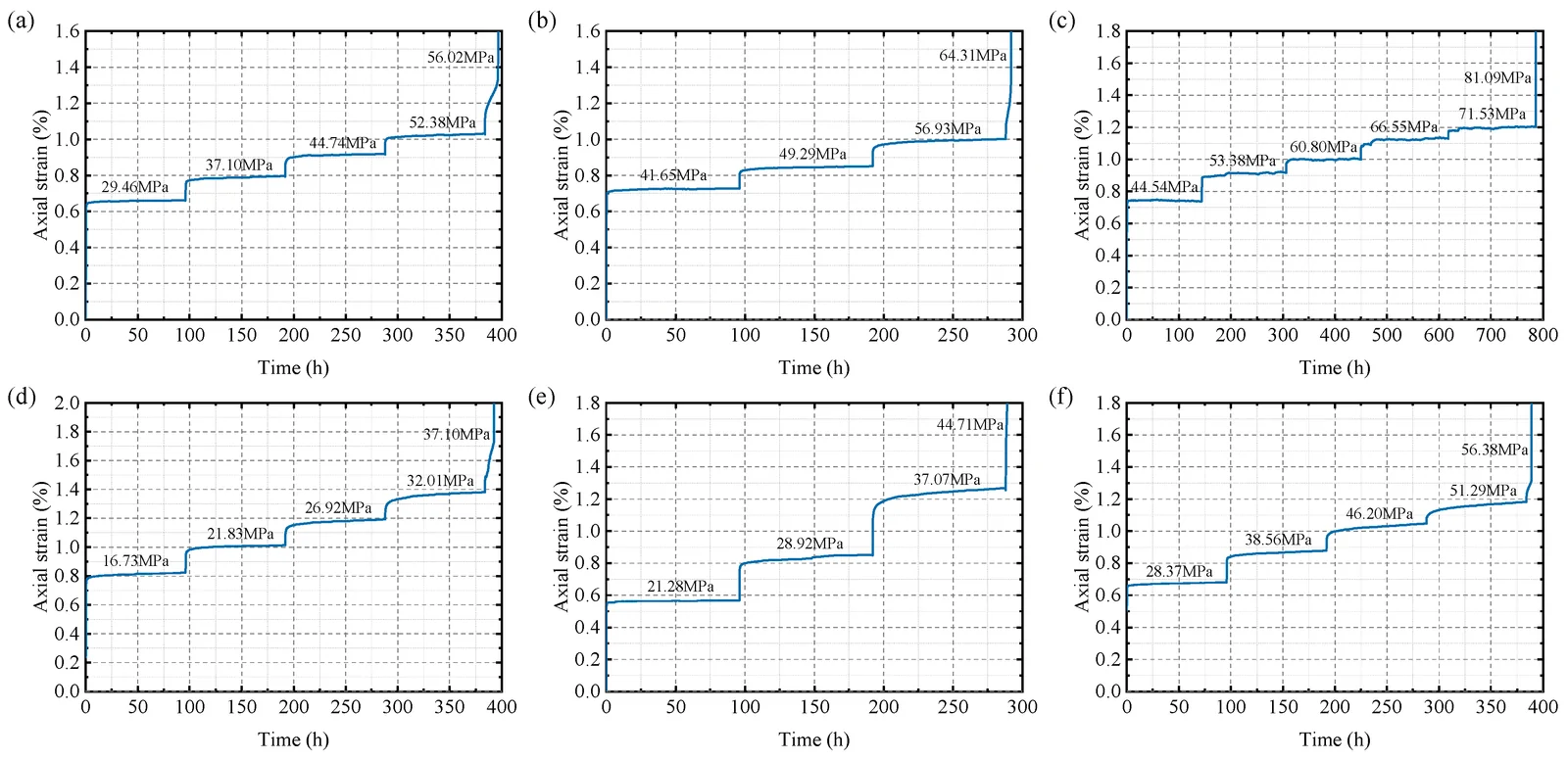
Full process curves of the triaxial compression creep test using argillaceous siltstone: (**a**) A-1 with *σ*_3_ = 4 MPa, (**b**) A-2 with *σ*_3_ = 6 MPa, (**c**) A-3 with *σ*_3_ = 8 MPa, (**d**) S-1 with *σ*_3_ = 4 MPa, (**e**) S-2 with *σ*_3_ = 6 MPa, (**f**) S-3 with *σ*_3_ = 8 MPa.

The full process curves of the triaxial compression creep test using argillaceous siltstone are shown in Figure 4, which shows that the creep characteristics of argillaceous siltstone are jointly affected by confining pressure and the water-bearing state. As the confining pressure increases from 4 MPa to 8 MPa, the axial stress level corresponding to the specimen entering the accelerating creep stage or undergoing failure increases. Taking the air-dried specimen as an example, the axial stress at creep failure increases from about 56.02 MPa to about 81.09 MPa, indicating that increasing confining pressure helps enhance the long-term bearing capacity of argillaceous siltstone.

Comparing air-dried and saturated specimens, it can be seen that water-wetting reduces the stress level corresponding to the creep failure of argillaceous siltstone. Under the same confining pressure, the axial stress at which saturated specimens enter the accelerating creep stage or undergo failure is lower than that of air-dried specimens. Taking a confining pressure of 8 MPa as an example, the air-dried specimen fails at 81.09 MPa, while the saturated specimen enters the failure stage at 56.38 MPa, indicating that an increase in water content reduces the long-term strength of argillaceous siltstone.

**Figure 5:**
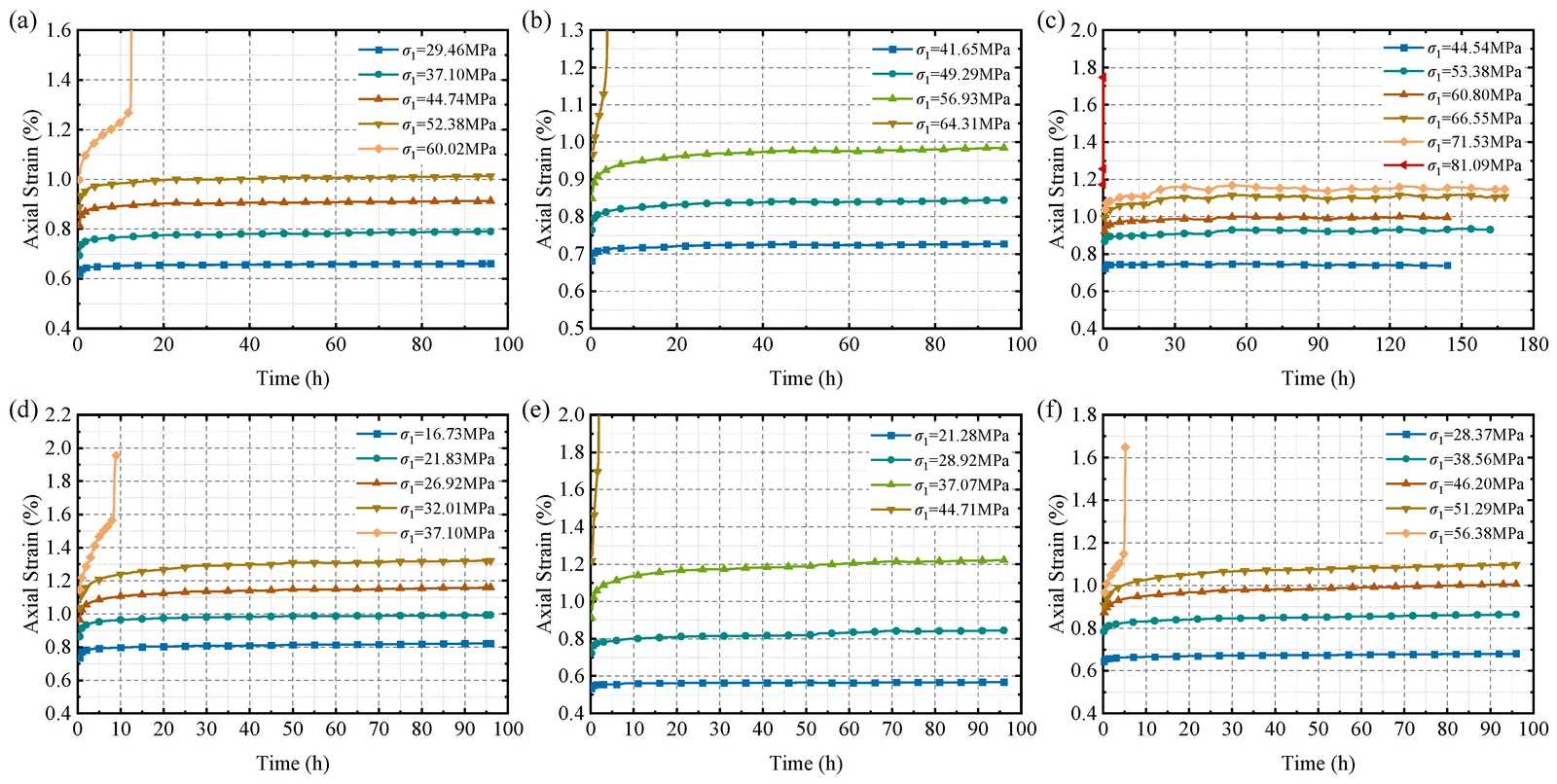
Axial strain–time curves at each load level: (**a**) A-1 with *σ*_3_ = 4 MPa, (**b**) A-2 with *σ*_3_ = 6 MPa, (**c**) A-3 with *σ*_3_ = 8 MPa, (**d**) S-1 with *σ*_3_ = 4 MPa, (**e**) S-2 with *σ*_3_ = 6 MPa, (**f**) S-3 with *σ*_3_ = 8 MPa.

The step-loading creep test curves for argillaceous siltstone were processed using Chen’s loading method [44] to obtain the axial strain–time curves under each load level, as shown in Figure 5. Chen’s loading method is a stepwise-loading and data-processing approach in which a single specimen is subjected to multistep loading; the measured total strain–time response is then decomposed into the individual creep curves for each load level. The axial creep characteristics of argillaceous siltstone are significantly affected by the stress level. Under each load level, the axial strain increases rapidly in the early stage of loading, then the growth rate gradually decreases and enters a relatively stable stage; under the failure load, the specimen undergoes accelerated deformation and eventually fails. From the perspective of strain rate, the axial strain rate is relatively large at the beginning of each loading stage, then decreases rapidly and gradually stabilizes. At low stress levels, the steady-state axial strain rate is small, and the axial creep deformation of the specimen develops slowly; as the axial stress increases, the steady-state axial strain rate generally increases, and the strain increment between creep curves at each level also increases, indicating that the time-dependent deformation of argillaceous siltstone is more obvious under high stress levels.

When loaded to the failure-level load, the axial strain rate begins to increase after an initial decrease or brief stabilization, and the specimen enters the accelerating creep stage. Comparing different water-bearing states, it can be seen that the creep deformation amplitude and steady-state strain rate of saturated argillaceous siltstone are generally higher than those of air-dried argillaceous siltstone under the same or similar stress levels, indicating that water-wetting increases the deformation rate of argillaceous siltstone in the steady-state creep stage and reduces its stability under long-term loading. Overall, the axial creep strain rate of argillaceous siltstone increases with increasing stress level, showing obvious accelerated growth under failure load; after saturation, both the steady-state strain rate and the creep deformation amplitude increase.

### 3.2. Creep Failure Characteristics

**Figure 6:**
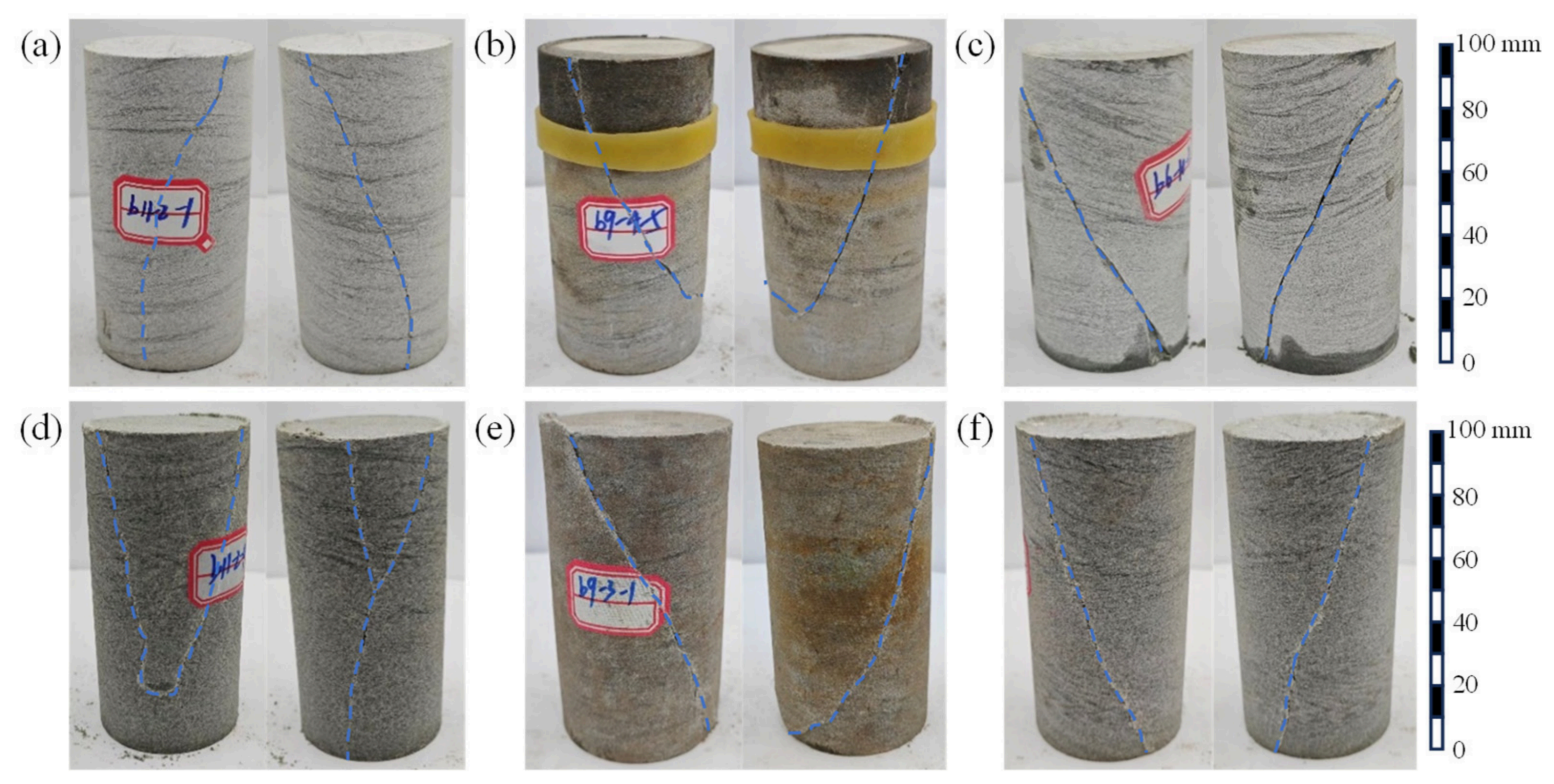
Final failure modes of argillaceous siltstone specimens after triaxial creep test: (**a**) A-1 with *σ*_3_ = 4 MPa, (**b**) A-2 with *σ*_3_ = 6 MPa, (**c**) A-3 with *σ*_3_ = 8 MPa, (**d**) S-1 with *σ*_3_ = 4 MPa, (**e**) S-2 with *σ*_3_ = 6 MPa, (**f**) S-3 with *σ*_3_ = 8 MPa.

The final failure modes of the argillaceous siltstone specimens in different water-bearing states after the triaxial creep test are shown in Figure 6. After triaxial creep, the argillaceous siltstone specimens mostly exhibit shear failure, with good connectivity of the main cracks. Air-dried specimens are clearly separated by a main crack. For saturated specimens, local fragmentation and spalling near the failure surface are more obvious, and the crack width is relatively larger, indicating that water-wetting increases the degree of local fragmentation near the failure surface of argillaceous siltstone. Failure morphology differs between different specimens. Specimen S-1 exhibits V-shaped shear failure characteristics, where the main fracture surface is not a single flat plane but is formed by the intersection of two oblique cracks, indicating that the failure path of this specimen is significantly affected by local structural differences. Specimens such as A-1, S-1, and S-2 exhibit oblique shear cracks as the main feature after failure, and lateral bulging is not obvious.

Overall, the triaxial creep failure of argillaceous siltstone is mainly shear failure, but there are differences in failure surface morphology, degree of local fragmentation, and lateral deformation characteristics among different water-bearing states and specimens. Water-wetting increases local fragmentation and spalling near the failure surface, while the structural differences in the specimens themselves also affect the final failure path.

## 4. Effect of Water-Induced Deterioration on Creep Mechanical Properties of Argillaceous Siltstone

In this section, we further analyze the influence of water-induced deterioration on the creep mechanical properties of argillaceous siltstone based on indicators such as the creep deformation amount, steady-state creep rate, and long-term strength.

### 4.1. Effect of Water-Induced Deterioration on Creep Deformation Proportion

**Figure 7:**
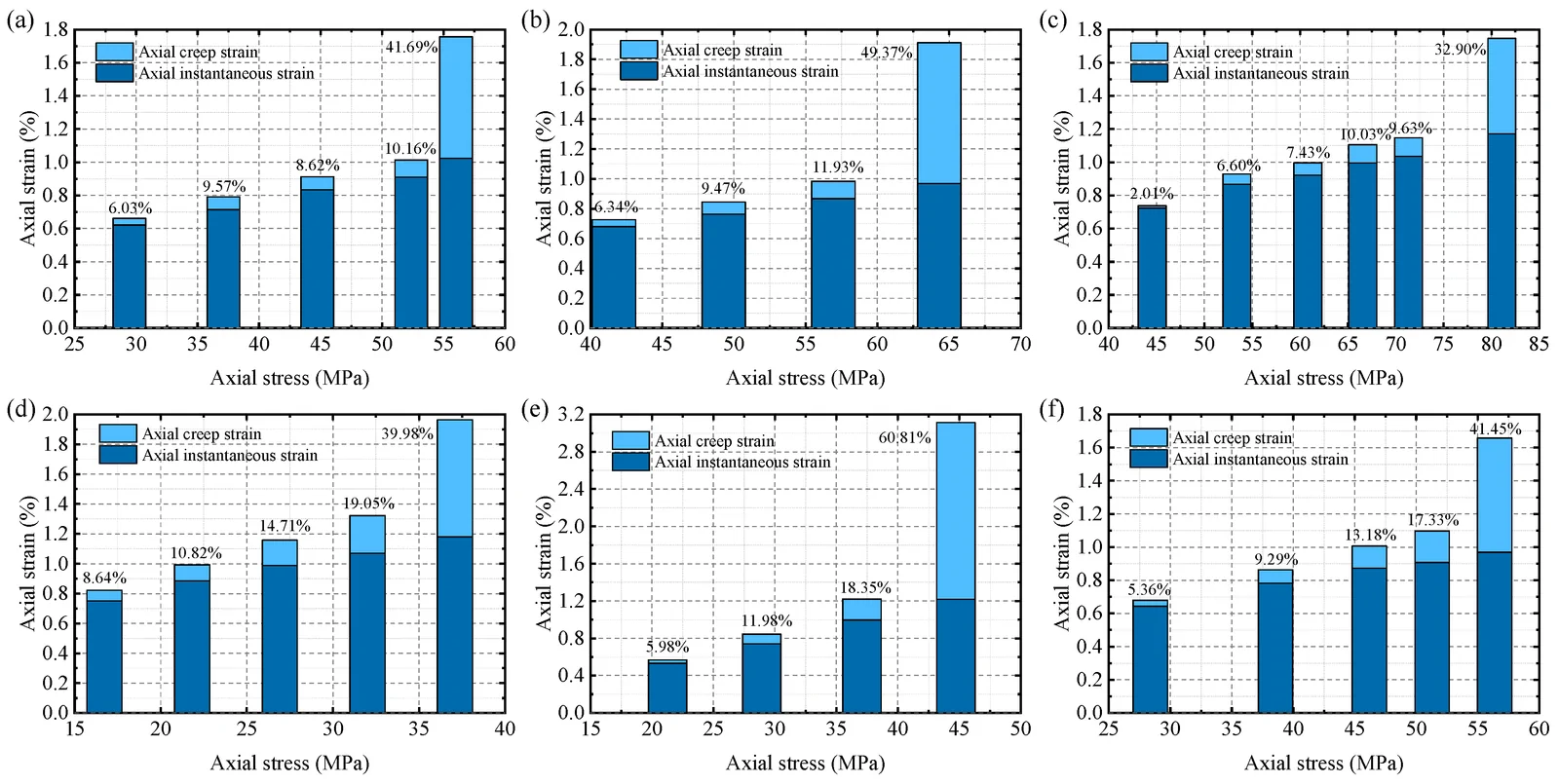
Bar chart of axial strain composition under different axial stresses: (**a**) A-1 with *σ*_3_ = 4 MPa, (**b**) A-2 with *σ*_3_ = 6 MPa, (**c**) A-3 with *σ*_3_ = 8 MPa, (**d**) S-1 with *σ*_3_ = 4 MPa, (**e**) S-2 with *σ*_3_ = 6 MPa, (**f**) S-3 with *σ*_3_ = 8 MPa.

The axial instantaneous strain and axial creep strain of specimens under each load level were statistically analyzed, and bar charts of axial strain composition under different axial stresses were plotted, as shown in Figure 7. The percentage shown above each bar in the figure represents the proportion of axial creep strain to the total axial strain. The total axial strain of each group of specimens generally increases with increasing axial stress, and the proportions of instantaneous strain and creep strain vary at different stress levels. At low to medium stress levels, the total axial strain is mainly composed of instantaneous strain, with a relatively small proportion of creep strain; under air-dried conditions, the creep strain proportion is usually about 2.01% to 11.93%, whereas under saturated conditions, it is about 5.36% to 19.05%. As the axial stress continues to increase and approaches the failure load, the creep strain increases significantly, and the creep proportion increases to 32.90% to 49.37% for air-dried specimens and to 39.98% to 60.81% for saturated specimens, indicating that the contribution of creep deformation to the total deformation of argillaceous siltstone increases under high stress levels, and that this contribution is more pronounced for the saturated specimens than for the air-dried ones.

Confining pressure has a certain restraining effect on the creep deformation of argillaceous siltstone. Under the same water-bearing state, as the confining pressure increases, the axial stress level at which the creep strain proportion significantly increases is raised accordingly. Taking the air-dried specimen as an example, this stress level increases from 56.02 MPa to 64.31 MPa and 81.09 MPa, indicating that enhanced lateral restraint helps delay the onset of the accelerating creep stage and improve the deformation stability of argillaceous siltstone under long-term loading. The water-bearing state also has a significant impact on the creep deformation of argillaceous siltstone. Under the same confining pressure conditions, the total strain and creep strain of saturated specimens are generally higher than those of air-dried specimens, and the proportion of creep strain to total strain also increases accordingly. For example, under a confining pressure of 4 MPa and an axial stress of 37.10 MPa, the creep proportion of the air-dried specimen is 9.57%, while that of the saturated specimen is 39.98%. This result indicates that water-wetting increases the creep deformation of argillaceous siltstone, enhances its contribution to total deformation, and reduces the bearing capacity of the specimen.

### 4.2. Effect of Water-Induced Deterioration on Instantaneous Elastic Modulus

**Figure 8:**
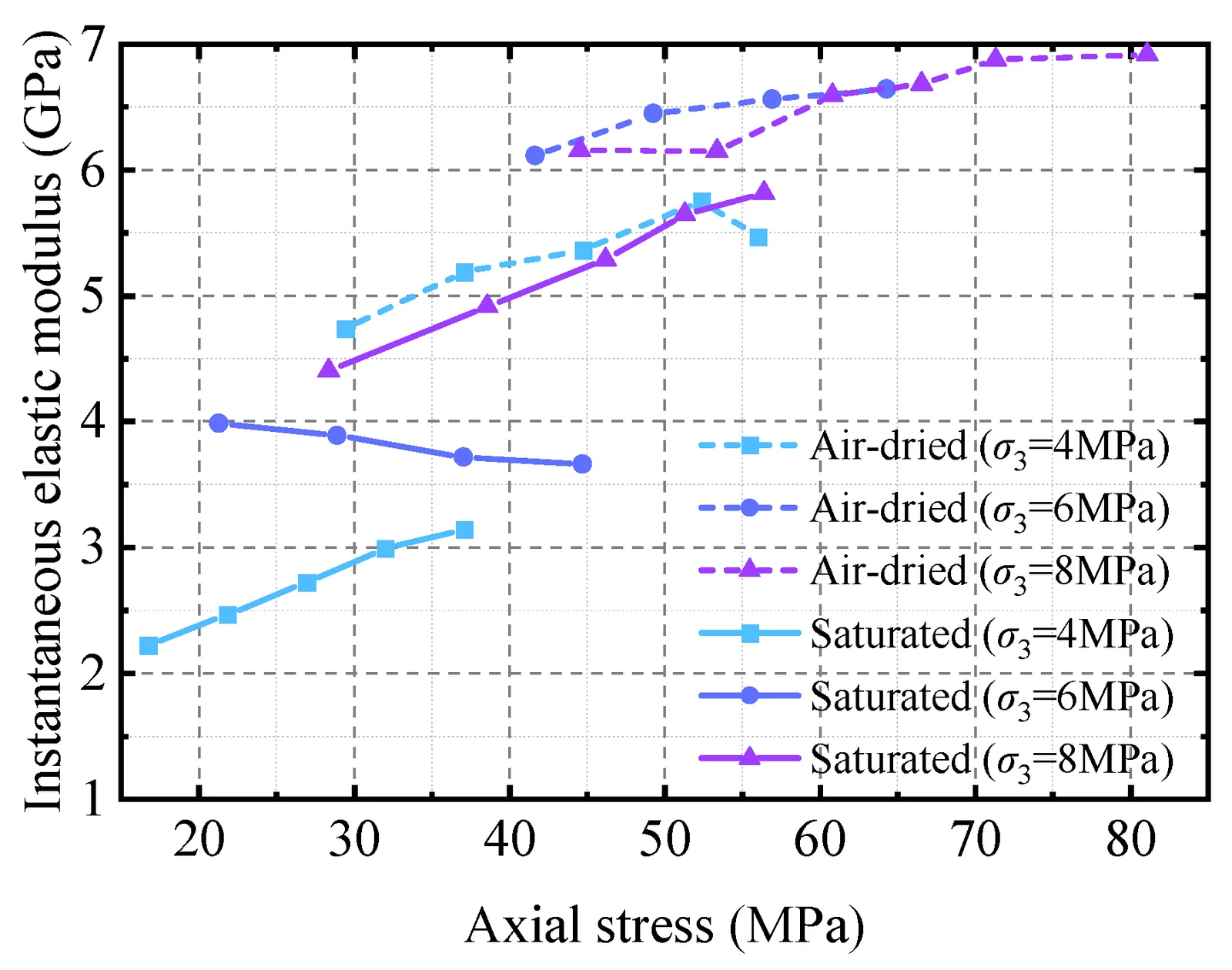
Variation in instantaneous elastic modulus with axial stress under different confining pressures.

Furthermore, the corresponding instantaneous elastic modulus was obtained based on the axial instantaneous strain at each loading stage, and its variation is shown in Figure 8. The instantaneous elastic modulus of argillaceous siltstone is jointly affected by the water-bearing state and confining pressure level. As the confining pressure increases, the instantaneous elastic modulus of the specimen generally increases, indicating that enhanced lateral restraint can effectively limit the instantaneous deformation of the specimen and improve the deformation resistance of argillaceous siltstone at the initial stage of loading. Under the same confining pressure condition, the instantaneous elastic modulus generally shows an upward trend with increasing axial stress, but the increase is not entirely consistent, sometimes showing slight fluctuations or tending to flatten.

In addition, water-wetting reduces the instantaneous elastic modulus of argillaceous siltstone. Under higher-confining-pressure conditions, saturated argillaceous siltstone can maintain a certain instantaneous elastic modulus, and no obvious softening trend appears with increasing axial stress; in contrast, under low-confining-pressure conditions, the effect of water-wetting on the instantaneous elastic modulus is more pronounced. The instantaneous elastic modulus of saturated specimens increases to a lesser extent with increasing axial stress, and the specimen under a confining pressure of 6 MPa even shows a slight decrease, indicating that argillaceous siltstone still has a certain bearing capacity under water-wetting action, but under low-confining-pressure conditions, the water-induced deterioration effect on argillaceous siltstone intensifies. Overall, the instantaneous elastic modulus of argillaceous siltstone mainly shows a pattern of increasing with increasing confining pressure and generally increasing with increasing axial stress, while water-wetting reduces its instantaneous elastic modulus.

### 4.3. Effect of Water-Induced Deterioration on Steady-State Creep Strain Rate

**Figure 9:**
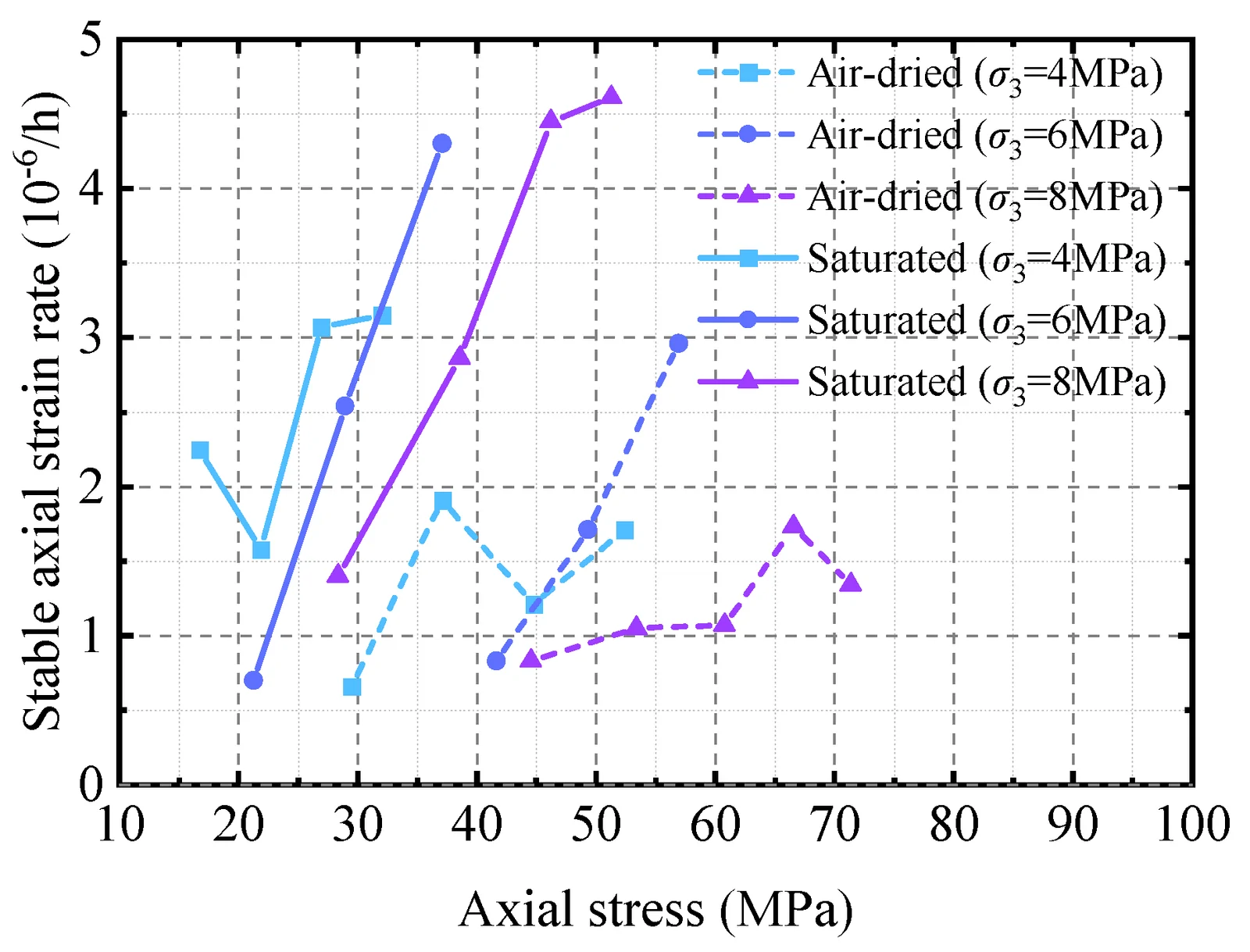
Variation in the axial strain rate in the steady-state creep stage with axial stress.

Figure 9 summarizes the axial strain rate of argillaceous siltstone in the steady-state creep stage. The axial strain rate of argillaceous siltstone in the steady-state creep stage is also jointly affected by axial stress, confining pressure, and the water-bearing state. Overall, the axial strain rate of each group of specimens in the steady-state creep stage generally shows an increasing trend with increasing axial stress, but there are significant differences in the growth amplitude under different water-bearing states. The axial strain rate of air-dried argillaceous siltstone in the steady-state creep stage is relatively low, showing a slow increase with increasing axial stress. For example, under a confining pressure of 4 MPa, it increases from 0.6 × 10^−6^/h to 1.9 × 10^−6^/h; under 6 MPa, from 0.8 × 10^−6^/h to 2.9 × 10^−6^/h; and under 8 MPa, from 0.8 × 10^−6^/h to 1.7 × 10^−6^/h. The axial strain rate of saturated specimens in the steady-state creep stage is relatively high, also showing a slow increase with increasing axial stress. For example, under a confining pressure of 4 MPa, it increases from 2.3 × 10^−6^/h to 3.1 × 10^−6^/h; under 6 MPa, from 0.7 × 10^−6^/h to 4.3 × 10^−6^/h; and under 8 MPa, from 1.4 × 10^−6^/h to 4.6 × 10^−6^/h. It demonstrates that under the same confining pressure conditions, the axial strain rate of saturated argillaceous siltstone in the steady-state creep stage is generally higher than that of air-dried argillaceous siltstone, indicating that water-wetting increases the axial strain rate of argillaceous siltstone in the steady-state creep stage. In addition, the influence of confining pressure on the axial strain rate in the steady-state creep stage is related to the water-bearing state. Under the air-dried state, the axial strain rate in the steady-state creep stage is generally lower under higher confining pressure conditions, indicating that confining pressure has a certain constraining effect on axial creep deformation; under the saturated state, although increasing confining pressure allows the specimen to withstand higher axial stress, the axial strain rate in the steady-state creep stage still increases significantly with increasing stress, indicating that water-wetting weakens the constraining effect of confining pressure on axial creep deformation.

In summary, the axial strain rate of argillaceous siltstone in the steady-state creep stage mainly shows an increase with increasing axial stress, and the axial strain rate of saturated specimens in the steady-state creep stage is generally higher than that of air-dried specimens. Water-wetting generally increases the axial strain rate of argillaceous siltstone in the steady-state creep stage.

### 4.4. Effect of Water-Induced Deterioration on Long-Term Strength

Long-term strength is an important concept in rock rheology research, reflecting the stress threshold at which rock transitions from steady-state creep to accelerating creep under long-term loading, and is directly related to the long-term stability of rock mass engineering. Long-term strength can be determined based on the morphological changes shown in isochronous curves; therefore, we adopt the isochronous curve method in this paper [20,21,22]. When the stress level is below the long-term strength, the distribution of isochronous curves at different times is relatively concentrated, the curve spacing is small, and the stress–strain relationship changes gently, indicating that the creep deformation of the specimen develops stably within this stress range. When the stress approaches or exceeds a certain critical level, the isochronous curves shift significantly toward the direction of increasing strain, the curve spacing increases, and the stress–strain relationship exhibits obvious nonlinear characteristics, indicating that the creep deformation of the specimen accelerates with time accumulation and gradually transitions from steady-state creep to accelerating creep. The stress level corresponding to this inflection point can be used as the basis for determining the long-term strength of the specimen. When the inflection point lies between two consecutive load levels, the long-term strength was obtained using linear interpolation of the axial stress.

**Figure 10:**
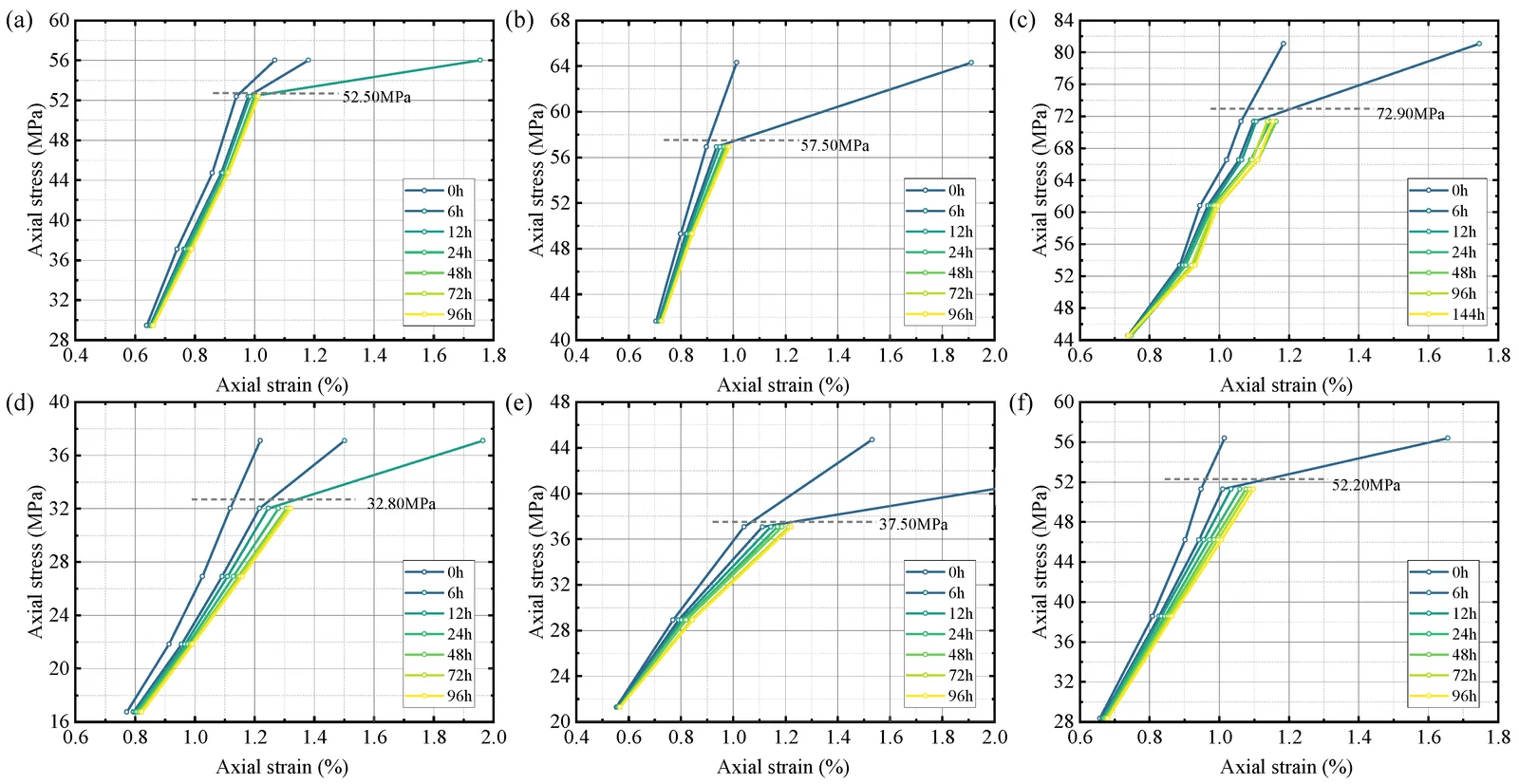
Isochronous axial stress–axial strain curves of argillaceous siltstone under triaxial compression creep: (**a**) A-1 with *σ*_3_ = 4 MPa, (**b**) A-2 with *σ*_3_ = 6 MPa, (**c**) A-3 with *σ*_3_ = 8 MPa, (**d**) S-1 with *σ*_3_ = 4 MPa, (**e**) S-2 with *σ*_3_ = 6 MPa, (**f**) S-3 with *σ*_3_ = 8 MPa.

The isochronous axial stress–axial strain curves of the argillaceous siltstone under triaxial compression creep testing are shown in Figure 10. For each specimen, isochronous curves at t = 0, 24, 48, 72, 96 h (and longer if available) were plotted. The isochronous curves for argillaceous siltstone generally exhibit clear stage characteristics, and the long-term strength determination point is easy to identify. The dispersion degree of the isochronous curves for argillaceous siltstone in the low-stress stage is relatively small; after entering a higher stress level, the turning point of the isochronous curves becomes more prominent, reflecting that the creep deformation of argillaceous siltstone accelerates with increasing stress. The turning point of the isochronous curves in the figure is relatively clear, indicating that the isochronous curve method has good applicability for determining the long-term strength of argillaceous siltstone. The results are as follows: the long-term strengths of air-dried argillaceous siltstone specimens under confining pressures of 4 MPa, 6 MPa, and 8 MPa are approximately 52.50 MPa, 57.50 MPa, and 72.90 MPa, respectively; under the saturated state, the long-term strengths decrease to 32.80 MPa, 37.50 MPa, and 52.20 MPa, respectively. Overall, the long-term strength of argillaceous siltstone increases with increasing confining pressure, indicating that confining pressure improves the long-term strength of argillaceous siltstone. In addition, under the same confining pressure, the long-term strength decreases significantly with increasing water content. The strength reductions in saturated specimens under confining pressures of 4 MPa, 6 MPa, and 8 MPa are approximately 37.5%, 34.8%, and 28.4%, respectively, indicating that water-wetting weakens the long-term bearing capacity of argillaceous siltstone, causing it to enter the unstable creep stage at a lower stress level. It also shows that as the confining pressure increases, the reduction in long-term strength caused by water content decreases, indicating that the increase in confining pressure has a certain compensatory effect on the long-term strength of argillaceous siltstone, which can partially mitigate the adverse effect of water-wetting action on long-term strength.

**Figure 11:**
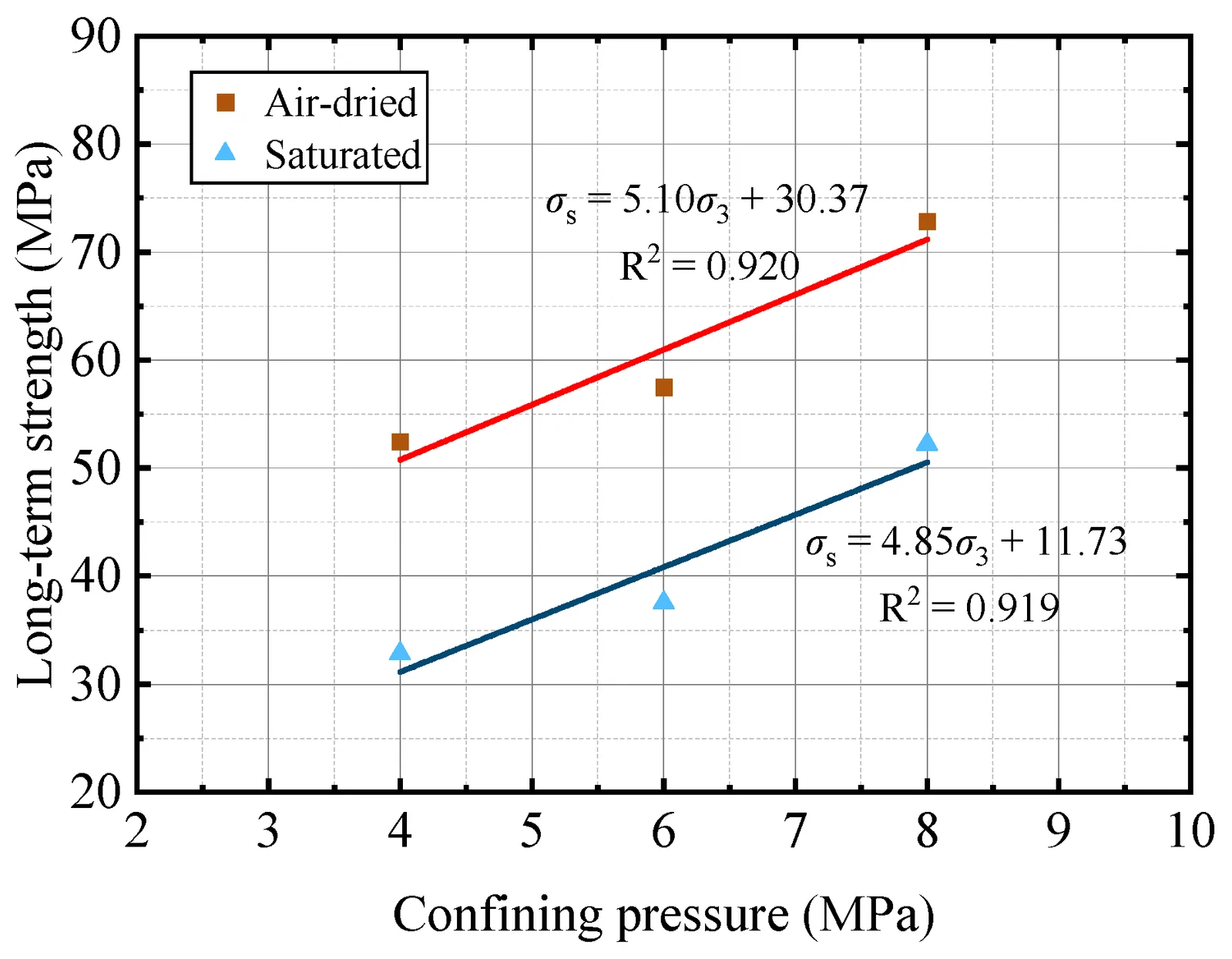
Relationship between long-term strength and confining pressure under different water-bearing states.

Based on the above long-term strength determination results, the relationship between long-term strength and confining pressure under different water-bearing states was fitted based on the Mohr–Coulomb strength criterion, as shown in Figure 11. Both the air-dried and saturated long-term strengths increase linearly with increasing confining pressure, indicating that increasing confining pressure enhances the long-term strength of argillaceous siltstone. From the fitting results, the coefficients of determination between long-term strength and confining pressure under different water-bearing states are all above 0.90, showing good linear correlation.

According to the fitting parameters of long-term strength, the long-term shear strength parameters under different water-bearing states can be further obtained, and the results are shown in Table 3, which shows that the long-term shear strength parameters of argillaceous siltstone are all affected by the water-bearing state. The internal friction angle of argillaceous siltstone decreases from 42.25° in the air-dried state to 41.15° in the saturated state, a relatively small change; meanwhile, the cohesion decreases from 6.72 MPa to 2.66 MPa, a substantial reduction. This suggests that water-induced deterioration likely weakens the intergranular cementation, leading to a reduction in cohesion, while the change in internal friction angle is relatively limited. It should be noted that the linear regression of long-term strength against confining pressure is based on only three data points; therefore, the estimated cohesion and internal friction angle are preliminary.

## 5. Creep Constitutive Model Based on Moisture–Stress Coupled Damage

### 5.1. Basic Component Model

The component constitutive model is an important theory for describing the characteristics of creep curves. The creep deformation characteristics of argillaceous siltstone demonstrate that when the stress does not exceed the long-term strength, the creep curves all have a decelerating creep stage and a steady-state creep stage, which is quite consistent with the Burgers model. When the stress exceeds the long-term strength, the creep curve transitions from viscoelastic to viscoplastic, requiring the further introduction of a viscoplastic component into the Burgers model.

**Figure 12:**
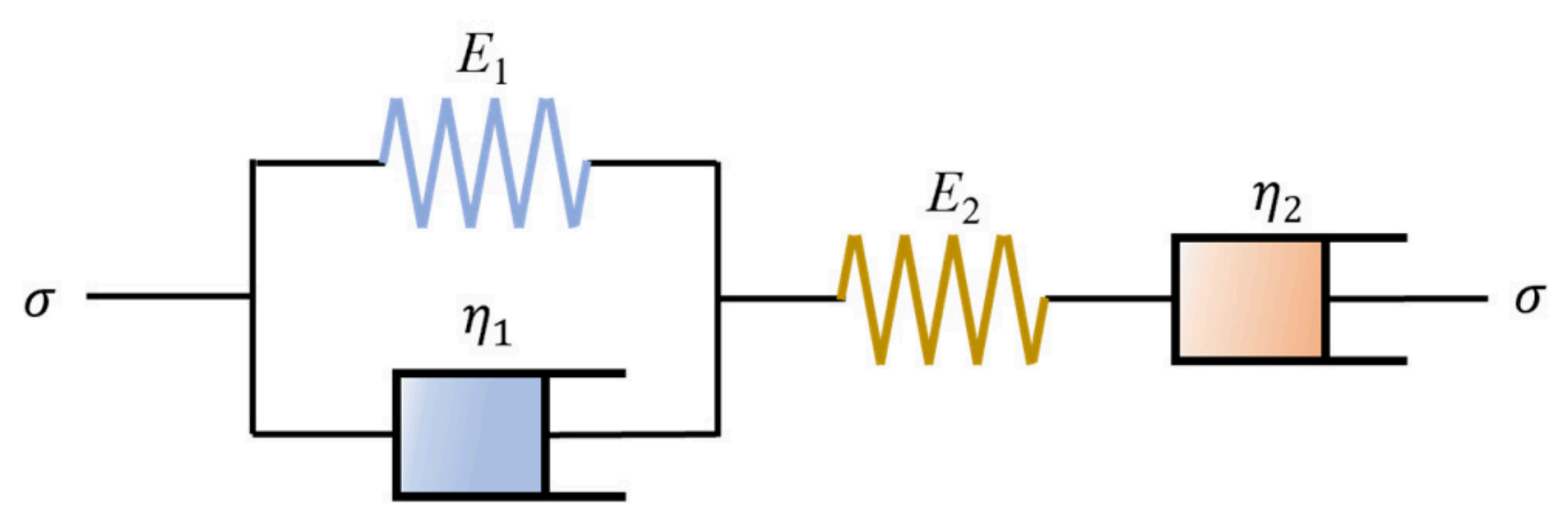
Schematic diagram of the Burgers model.

The Burgers model consists of a Kelvin component and a Maxwell component connected in series, as shown in Figure 12, and its constitutive equation is

(1) $$\varepsilon =\frac{\sigma }{E_{1}}\left[1-\exp\left(-\frac{E_{1}}{\eta_{1}}t\right)\right]+\frac{\sigma }{E_{2}}+\frac{\sigma }{\eta_{2}}$$

where *ε* is the creep strain, *σ* is the stress, *t* is time, *E*_1_ and *E*_2_ are the elastic moduli of the spring elements in the Maxwell component and Kelvin component, respectively, and *η*_1_ and *η*_2_ are the viscosity coefficients of the dashpots in the Maxwell component and Kelvin component, respectively.

**Figure 13:**
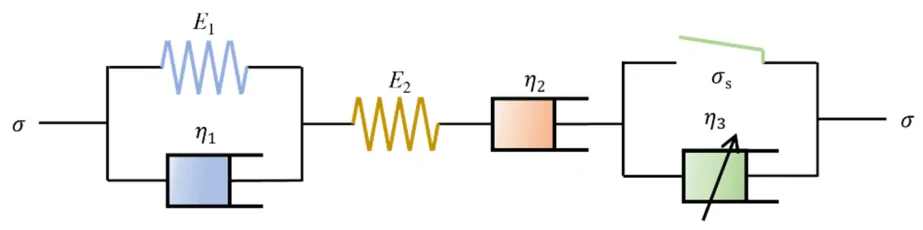
Schematic diagram of the improved nonlinear viscoelastic–plastic creep model.

Regarding the viscoplasticity of argillaceous siltstone, this study introduces a nonlinear viscoplastic component to perform a nonlinear improvement on the Burgers model. The obtained model is shown in Figure 13, and the constitutive equations of the nonlinear viscoplastic component and the improved model are

(2) $${\dot{\varepsilon }}_{3}=\frac{\langle \sigma -\sigma_{s}\rangle }{\eta_{3}}\exp\left(\mathrm{nt}\right)$$

(3) $$\varepsilon =\frac{\sigma }{E_{1}}\left[1-\exp\left(-\frac{E_{1}}{\eta_{1}}t\right)\right]+\frac{\sigma }{E_{2}}+\frac{\sigma }{\eta_{2}}+\frac{\langle \sigma -\sigma_{s}\rangle }{\eta_{3}n}\left[\exp\left(\mathrm{nt}\right)-1\right]$$

where *η*_3_ is the viscosity coefficient of the nonlinear viscoplastic component, *σ*_s_ is the long-term strength, and *n* is a coefficient related to material properties and stress state; 〈〉 is the Macaulay bracket, the physical meaning of which is the definition of the plastic yield condition; i.e., this component is activated only when the stress exceeds the long-term strength, producing irreversible deformation.

In addition, the long-term deterioration of argillaceous siltstone is not caused by a single factor but is the result of the combined influence of water-wetting and stress level. To more realistically reflect this physical process, this paper introduces a moisture–stress coupled damage evolution mechanism. First, for the deterioration of argillaceous siltstone caused by water intrusion, a water-induced damage factor *D_ω_* based on water content is defined as

*D_ω_* = 1 − exp(−*βω*) (4)

where ω is the water content, and *β* is the water-induced damage evolution coefficient characterizing the sensitivity of the material to water. The exponential form is motivated by continuum damage mechanics [39,40,41,42] and has been widely used to represent progressive material degradation.

Secondly, when the stress exceeds the long-term strength, a stress damage factor *D*_s,*t*_ is introduced to characterize the deterioration of material parameters over time under high stress conditions, and its expression is

*D*_s,*t*_ = 1 − exp[−*α*〈*σ* − *σ*_s_〉*t*] (5)

where α is a characteristic parameter controlling the rate of stress damage accumulation.

Considering the combined effect of water-induced damage and stress damage, the composite damage factor is expressed as

*D* = 1 − (1 − *D*_s,*t*_)(1 − *D_ω_*) = 1 − exp[−*α*〈*σ* − *σ*_s_〉*t* − *βω*] (6)

When the water content ω = 0, the damage factor degenerates to *D*_s,*t*_; when the stress does not exceed the long-term strength of the rock, the damage factor degenerates to *D_ω_*; and when the rock is in a dry state and the stress is below the long-term strength, the damage is zero.

Based on this moisture–stress coupled damage factor, assuming that the viscoelastic parameters of the creep model are reduced as the damage factor increases, the viscoelastic parameters considering composite damage can be obtained as follows:

(7) $$\begin{array}{c} {E_{1}}^{\prime }=E_{1}\exp\left[-\beta \omega -\alpha \langle \sigma -\sigma_{s}\rangle t\right]\\ {E_{2}}^{\prime }=E_{2}\exp\left[-\beta \omega -\alpha \langle \sigma -\sigma_{s}\rangle t\right]\\ {\eta_{1}}^{\prime }=\eta_{1}\exp\left[-\beta \omega -\alpha \langle \sigma -\sigma_{s}\rangle t\right]\\ {\eta_{2}}^{\prime }=\eta_{2}\exp\left[-\beta \omega -\alpha \langle \sigma -\sigma_{s}\rangle t\right] \end{array}$$

Combining Equations (3) and (7), the nonlinear viscoelastic–plastic creep constitutive model considering moisture–stress coupled time-dependent damage is obtained as follows:

(8) $$\begin{array}{c} \varepsilon =\frac{\sigma }{\eta_{1}A+E_{1}}\left[\exp\left(\mathrm{At}+\beta \omega \right)-\exp\left(\beta \omega -\frac{E_{1}}{\eta_{1}}t\right)\right]+\frac{\sigma }{E_{2}}\exp\left(\beta \omega +\mathrm{At}\right)\\ +\frac{\sigma }{\eta_{2}}\mathrm{texp}\left(\beta \omega +\mathrm{At}\right)+\frac{\langle \sigma -\sigma_{s}\rangle }{\eta_{3}n}\left[\exp\left(\mathrm{nt}\right)-1\right] \end{array}$$

where *A* = *α*〈*σ* − *σ*_s_〉. Because of the Macaulay bracket, the stress damage term vanishes when *σ* ≤ *σ*_s_, so the composite damage factor reduces to *βω*.

Furthermore, Equation (8) can be expressed as

(9) $$\varepsilon =\left\{\begin{array}{cc} \begin{array}{l} \frac{\sigma }{\eta_{1}A+E_{1}}\left[\exp\left(\mathrm{At}+\beta \omega \right)-\exp\left(\beta \omega -\frac{E_{1}}{\eta_{1}}t\right)\right]+\frac{\sigma }{E_{2}}\exp\left(\beta \omega +\mathrm{At}\right)\\ +\frac{\sigma }{\eta_{2}}\mathrm{texp}\left(\beta \omega +\mathrm{At}\right) \end{array} & \sigma \leq \sigma_{s}\\ \begin{array}{l} \frac{\sigma }{\eta_{1}A+E_{1}}\left[\exp\left(\mathrm{At}+\beta \omega \right)-\exp\left(\beta \omega -\frac{E_{1}}{\eta_{1}}t\right)\right]+\frac{\sigma }{E_{2}}\exp\left(\beta \omega +\mathrm{At}\right)\\ +\frac{\sigma }{\eta_{2}}\mathrm{texp}\left(\beta \omega +\mathrm{At}\right)+\frac{\langle \sigma -\sigma_{s}\rangle }{\eta_{3}n}\left[\exp\left(\mathrm{nt}\right)-1\right] \end{array} & \sigma >\sigma_{s} \end{array}\right.$$

### 5.2. Three-Dimensional Extension of the Creep Constitutive Model

In practical engineering, rocks are often in a complex three-dimensional in situ stress environment. To make the established damage creep model conform to the actual engineering situation, the one-dimensional model needs to be extended to three-dimensional stress conditions.

According to the assumption of constant bulk modulus, the stress tensor in three-dimensional stress space can be decomposed into a spherical stress tensor and a deviatoric stress tensor as follows:

***σ**_ij_* = *σ*_m_***δ**_ij_* + ***S**_ij_* (10)

where ***σ**_ij_* is the total stress tensor, *σ*_m_ = *σ_ii_*/3 is the spherical stress scalar, ***S**_ij_* is the deviatoric stress tensor, and ***δ**_ij_* is the Kronecker delta.

Correspondingly, the rock strain can also be decomposed into spherical strain and deviatoric strain components as follows:

(11) $$\varepsilon_{\mathrm{ij}}^{\mathrm{tol}}=\varepsilon_{m}\delta_{\mathrm{ij}}+e_{\mathrm{ij}}$$

where ***ε**_ij_* is the total strain tensor, *ε*_m_ = *ε_ii_*/3 is the spherical strain scalar, and ***e**_ij_* is the deviatoric strain tensor.

Under the assumption of a constant bulk modulus, it is considered that the viscoelastic creep of rock is mainly caused by deviatoric stress. Combined with the creep constitutive model established in the previous section, the creep strain of rock under load can be expressed as

(12) $$\varepsilon_{\mathrm{ij}}^{\mathrm{tol}}=\varepsilon_{m}\delta_{\mathrm{ij}}+e_{\mathrm{ij}}=\frac{\sigma_{m}\delta_{\mathrm{ij}}}{3{K_{2}}^{\prime }}+\frac{S_{\mathrm{ij}}}{2{G_{2}}^{\prime }}+\frac{S_{\mathrm{ij}}}{2{H_{2}}^{\prime }}t+e_{\mathrm{ij}}^{K}\left(S_{\mathrm{ij}}\right)+\varepsilon_{\mathrm{ij}}^{B}\left(S_{\mathrm{ij}}\right)$$

where $e_{\mathrm{ij}}^{K}\left(S_{\mathrm{ij}}\right)$ and $\varepsilon_{\mathrm{ij}}^{B}\left(S_{\mathrm{ij}}\right)$ are the strain tensors corresponding to the Kelvin component and Bingham component in three-dimensional strain space, respectively.

Before extending the one-dimensional constitutive model to three dimensions, it should be noted that in the Bingham component equation, the one-dimensional overstress scalar 〈*σ* − *σ*_s_〉 cannot be directly applied to calculating three-dimensional viscoplastic strain. To this end, according to the concept of flow potential in plasticity theory, the Perzyna equation [45] is introduced as follows:

(13) $$\langle \sigma -\sigma_{s}\rangle ={\langle \frac{F}{F_{0}}\rangle }^{m}\frac{\partial Q}{\partial \sigma_{\mathrm{ij}}}$$

where *F* is the yield function; *F*_0_ is a constant used to adjust the dimension, which can be taken as 1; for rock materials, *m* = 1; and *Q* is the plastic potential function.

Based on the above results, it is assumed that the plastic deformation behavior of argillaceous siltstone obeys the Mohr–Coulomb strength criterion as follows:

For shear yielding

(14) $$\left\{\begin{array}{l} F=\sigma_{1}-\frac{1+\mathrm{sin\varphi}}{1-\mathrm{sin\varphi}}\sigma_{3}-\frac{2\mathrm{ccos\varphi}}{1-\mathrm{sin\varphi}}\\ Q=\sigma_{1}-\frac{1+\mathrm{sin\psi}}{1-\mathrm{sin\psi}}\sigma_{3} \end{array}\right.$$

For tensile yielding

(15) $$\left\{\begin{array}{l} F=R_{t}-\sigma_{3}\\ Q=-\sigma_{3} \end{array}\right.$$

where *c*, *φ*, and *ψ* are the long-term cohesion, long-term internal friction angle, and long-term dilatancy angle of the rock during creep, respectively; *R*_t_ is the long-term tensile strength of the rock.

To simplify the equations, it is assumed that the three-dimensional elastic parameters directly follow the damage law specified in Equation (7). The 3D transition is based on the following constant-bulk-modulus assumption: the spherical part produces only elastic volumetric strain, whereas the deviatoric part drives viscoelastic creep, and the viscoplastic component is extended to 3D through the Perzyna overstress theory using the Mohr–Coulomb yield function *F*. Meanwhile, the overstress scalar ⟨*σ* − *σ_s_*⟩ in the three-dimensional stress damage factor *D_s_*_,*t*_ is replaced by ⟨*σ*_1_ − *σ_s_*⟩. Because the Mohr–Coulomb shear yield function is identically *F* = *σ*_1_ − *σ_s_*(*σ*_3_), the damage overstress ⟨*σ*_1_ − *σ_s_*⟩ is exactly ⟨*F*⟩; hence, the damage is driven by the same overstress measure that governs viscoplastic yielding and is activated only when the stress state exceeds the long-term Mohr–Coulomb yield surface.

Furthermore, it is assumed that the relationship between *σ_s_* and confining pressure *σ*_3_ follows

(16) $$\sigma_{s}=\frac{1+\sin\varphi }{1-\sin\varphi }\sigma_{3}+\frac{2c\cos\varphi }{1-\sin\varphi }$$

Therefore,

(17) $${\dot{\varepsilon }}_{\mathrm{ij}}^{B}=\frac{\exp\left(\mathrm{nt}\right)}{2H_{3}}\langle F\rangle \frac{\partial Q}{\partial \sigma_{\mathrm{ij}}}$$

Combining Equations (9), (12), and (17), we have

(18) $$\varepsilon_{\mathrm{ij}}^{M}=\left(\frac{\sigma_{m}\delta_{\mathrm{ij}}}{3K_{2}}+\frac{S_{\mathrm{ij}}}{2G_{2}}\right)\exp\left(\beta \omega +\mathrm{At}\right)+\frac{S_{\mathrm{ij}}}{2H_{2}}t\exp\left(\beta \omega +\mathrm{At}\right)$$

(19) $$e_{\mathrm{ij}}^{K}=\frac{S_{\mathrm{ij}}}{2H_{1}A+2G_{1}}\left[\exp\left(\beta \omega +\mathrm{At}\right)-\exp\left(\beta \omega -\frac{G_{1}}{H_{1}}t\right)\right]$$

(20) $$\varepsilon_{\mathrm{ij}}^{B}=\frac{\langle F\rangle }{2H_{3}n}\frac{\partial Q}{\partial \sigma_{\mathrm{ij}}}\left[\exp\left(\mathrm{nt}\right)-1\right]$$

where *K*_1_, *G*_1_, and *H*_1_ are the initial bulk modulus, shear modulus, and shear viscosity coefficient of the Kelvin component in three-dimensional space, respectively; *K*_2_, *G*_2_, and *H*_2_ are the initial bulk modulus, shear modulus, and shear viscosity coefficient of the Maxwell component in three-dimensional space, respectively; and *H*_3_ is the initial shear viscosity coefficient of the Bingham component in three-dimensional space.

Thus, the constitutive relation of the damage creep model under a three-dimensional stress state is obtained as follows:

(21) $$\begin{array}{cc} \varepsilon_{\mathrm{ij}}^{\mathrm{tol}}= & \left\{\begin{array}{c} \left(\frac{\sigma_{m}\delta_{\mathrm{ij}}}{3K_{2}}+\frac{S_{\mathrm{ij}}}{2G_{2}}\right)\exp\left(\beta \omega +\mathrm{At}\right)+\frac{S_{\mathrm{ij}}}{2H_{2}}t\exp\left(\beta \omega +\mathrm{At}\right)\\ +\frac{S_{\mathrm{ij}}}{2H_{1}A+2G_{1}}\left[\exp\left(\beta \omega +\mathrm{At}\right)-\exp\left(\beta \omega -\frac{G_{1}}{H_{1}}t\right)\right]\\ +\frac{\langle F\rangle }{2H_{3}n}\frac{\partial Q}{\partial \sigma_{\mathrm{ij}}}\left[\exp\left(\mathrm{nt}\right)-1\right] \end{array}\right. \end{array}$$

### 5.3. Parameter Identification of Creep Constitutive Model for Argillaceous Siltstone

In this section, the Levenberg–Marquardt (L-M) nonlinear regression method is used to fit and determine the creep constitutive model parameters for argillaceous siltstone. For the free model parameters, the initial values and parameter constraints were generated automatically by the fitting procedure, and no manual lower or upper bounds were imposed on the free parameters. Parameters that are independently known or prescribed were held fixed rather than estimated—for example, the long-term strength σ_s_ was taken from the isochronous curve method, and the initial strain, the water-damage multiplier, and the applied stress level were fixed. Because the water-damage parameter *β* is a material parameter common to all specimens, it was not estimated level by level; instead, β was first identified by globally fitting the initial instantaneous strain–stress data of all specimens with different water-bearing states, which yielded a single constant *β* = 17.228. In the subsequent creep parameter identification, *β* was held fixed at this value, and only the remaining creep parameters were identified. The objective (error) function minimized by the L–M algorithm is the unweighted residual sum of squares (SSR) between the measured and predicted axial strains. The convergence criterion was that the iteration terminated when the SSR tolerance reached 1 × 10^−9^.

During the parameter inversion process, if the three-dimensional tensor constitutive equation is directly used for global fitting, the fitting stability may be affected by the large number of parameters, initial value selection, and parameter correlations. To facilitate parameter identification, this study processes the model expression piecewise according to the stress stage of the specimen.

(1) When the specimen is in the low-stress stage and has not entered the accelerating creep stage, the model mainly describes the viscoelastic decelerating creep and steady-state creep process as follows:

(22) $$\varepsilon_{1}(t)=\varepsilon_{1}^{0}+\frac{\sigma_{1}-\sigma_{3}}{3G_{1}}\exp(\beta \omega )\left[1-\exp\left(-\frac{G_{1}}{H_{1}}t\right)\right]+\frac{\sigma_{1}-\sigma_{3}}{3H_{2}}\exp(\beta \omega )t$$

(2) When the specimen is in the high-stress stage and exhibits obvious accelerating creep characteristics, the model further introduces the nonlinear viscoplastic term and damage correction term as follows:

(23) $$\varepsilon_{1}=\left\{\begin{array}{l} \varepsilon_{1}^{0}\exp\left(\mathrm{At}\right)+\frac{F}{2H_{3}n}\left[\exp\left(\mathrm{nt}\right)-1\right]+\frac{\sigma_{1}-\sigma_{3}}{3H_{2}}t\exp(\beta \omega +\mathrm{At})\\ +\frac{\sigma_{1}-\sigma_{3}}{3\left(H_{1}A+G_{1}\right)}\exp\left(\beta \omega \right)\left[\exp\left(\mathrm{At}\right)-\exp\left(-\frac{G_{1}}{H_{1}}t\right)\right] \end{array}\right.$$

where

(24) $$\varepsilon_{1}^{0}=\left(\frac{\sigma_{1}+2\sigma_{3}}{9K_{2}}+\frac{\sigma_{1}-\sigma_{3}}{3G_{2}}\right)\exp\left(\beta \omega \right)$$

It should be emphasized that this piecewise treatment is not merely a numerical convenience but also effectively mitigates the risk of overfitting associated with the relatively large nominal number of model parameters. Because the nonlinear viscoplastic component and the stress-damage term are activated only when *σ* > *σ*_s_ through the Macaulay bracket, the parameters *α*, *H*_3_, and *n* are estimated exclusively at the accelerating levels, whereas at the viscoelastic levels where *σ* ≤ *σ*_s_, only the viscoelastic parameters (*G*_1_, *H*_1_, G_2,_ and *H*_2_) are identified, with *β* fixed at 17.228 and the water-damage factor determined from the measured water content. Consequently, the number of free parameters simultaneously estimated at any single loading level is substantially smaller than the nominal total, and the additional parameters are introduced only where the underlying physics requires them.

**Figure 14:**
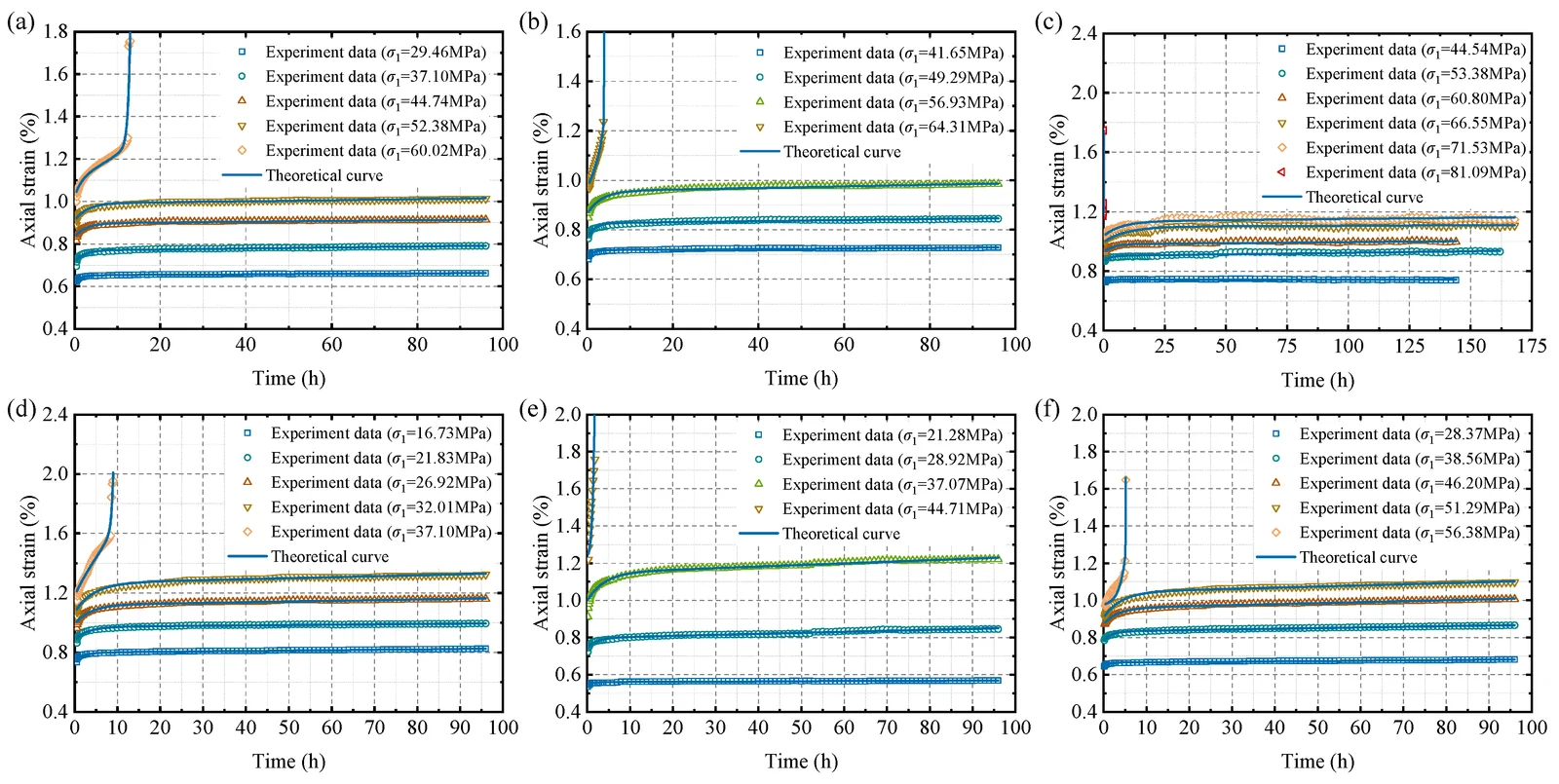
Comparison between model fitting curves and test data of argillaceous siltstone triaxial creep: (**a**) A-1 with *σ*_3_ = 4 MPa, (**b**) A-2 with *σ*_3_ = 6 MPa, (**c**) A-3 with *σ*_3_ = 8 MPa, (**d**) S-1 with *σ*_3_ = 4 MPa, (**e**) S-2 with *σ*_3_ = 6 MPa, (**f**) S-3 with *σ*_3_ = 8 MPa.

Based on the above three-dimensional damage creep model, parameters were identified in the triaxial step-loading creep test data for argillaceous siltstone under different water-bearing states and confining pressures. The comparison between the fitted curves and the measured data is shown in Figure 14, which shows that the fitted curves are in good agreement with the experiment data overall, with the coefficient of determination *R*^2^ of most tests greater than 0.90. The model can describe the decelerating creep and steady-state creep processes of argillaceous siltstone under various deviatoric stress levels well; for the accelerating creep deformation occurring in the high-deviatoric-stress stage, reasonable fitting results can also be obtained. This indicates that the three-dimensional damage creep model established in this study has good applicability to the triaxial creep deformation of argillaceous siltstone.

The identified constitutive model parameters are shown in Table 4. The creep parameters of argillaceous siltstone under different confining pressures and water-bearing states exhibit significant differences. Regarding the effect of confining pressure, under the same water-bearing state, the specimen can withstand a higher axial stress level when the confining pressure increases, indicating that lateral restraint improves the long-term bearing capacity of argillaceous siltstone. Among the model parameters, the shear modulus and viscosity coefficients reflecting the deformation characteristics of the steady-state creep stage generally increase with increasing confining pressure. For example, the modulus and viscosity coefficients under a confining pressure of 8 MPa are generally higher than those under a confining pressure of 4 MPa. This result indicates that increasing confining pressure can reduce the creep deformation of argillaceous siltstone to a certain extent and enhance its ability to resist long-term deformation.

Regarding the effect of the water-bearing state, after the water content increases, the long-term strength and some viscoelastic parameters of argillaceous siltstone generally decrease. Compared with low-water-content specimens, the parameters *G*_2_, *K*_2_, etc., of high-water-content specimens under similar confining pressures are generally lower, indicating that water-wetting weakens the deformation resistance of argillaceous siltstone during creep. In addition, some parameters exhibit certain regularities with changing stress levels. For example, *G*_2_ and *K*_2_ generally tend to increase with increasing axial stress, indicating that argillaceous siltstone still exhibits a certain compaction effect during the low- and medium-stress stages. In contrast, viscosity coefficients such as *H*_2_ fluctuate considerably under different stress levels, especially near the failure load, indicating that parameters in the high-stress stage are significantly affected by nonlinear accelerating creep, and the influencing mechanism is relatively complex. *G*_1_ and *H*_1_ also show a certain degree of dispersion, suggesting that the parameters of the decelerating creep stage are sensitive to the initial deformation upon loading, the duration of load holding, and specimen differences. When the stress approaches or exceeds the long-term strength, the nonlinear viscoplastic term in the model begins to participate in the fitting, and the relevant parameters *α*, *H*_3_, and *n* are used to describe the accelerating creep characteristics in the high-stress stage. The results show that these parameters vary greatly under different confining pressures and water-bearing states. Therefore, such parameters are mainly used to characterize the curve shape of the accelerating stage and should not be used alone as the sole basis for judging changes in material properties. It is worth noting that extremely large values occur in the accelerating creep stage for certain specimens; they arise because the exponential term exp(*nt*) dominates and the viscosity coefficient must compensate to keep the strain rate finite. Such extreme values indicate that the accelerating phase is very short.

Overall, increasing confining pressure enhances the long-term bearing capacity and deformation resistance of argillaceous siltstone, while increasing the water content reduces its long-term strength and some viscoelastic parameters. Under triaxial-confining-pressure conditions, argillaceous siltstone still maintains a relatively high modulus level and long-term bearing capacity, but water-wetting still increases its steady-state creep rate and enhances its long-term deformation capability.

### 5.4. Model Comparison

To quantitatively evaluate the advancement of the proposed nonlinear viscoelastic–plastic creep model, a direct comparison with the classical Burgers model was conducted for every loading level of all six specimens, with the statistical indicators including coefficient of determination *R*^2^, root mean square error *RMSE*, Akaike information criterion *AIC*, and Bayesian information criterion *BIC* summarized in Table 5. Both models were evaluated on identical datasets for each loading level, and all the statistical indicators were computed from the dimensionless axial strain. The *AIC* and *BIC* indicators are calculated as follows:

(25) $$\left\{\begin{array}{l} \mathrm{AIC}=N\ln({\mathrm{RMSE}}^{2})+2k\\ \mathrm{BIC}=N\ln({\mathrm{RMSE}}^{2})+k\ln(N) \end{array}\right.$$

where *N* is the number of data points used in the fit, and *k* is the number of free parameters.

It can be observed from Table 5 that, for the loading levels where the deviatoric stress remains below the long-term strength, both models achieve very close fitting accuracies. For example, for the saturated specimen S-3 at deviatoric stresses of 20.37, 30.56, 38.20, and 43.29 MPa, the *R*^2^ values of the Burgers model are 0.982, 0.989, 0.991, and 0.991, while those of the proposed model are 0.978, 0.983, 0.991, and 0.990, with the *RMSE* differing by less than 0.0005% in most cases. This consistency is expected because, when the stress does not exceed the long-term strength, the Macaulay bracket in the proposed model vanishes and the model degenerates to a Burgers-type formulation with parameters scaled according to the constant water-damage factor. Because both models are compared on identical datasets with the unified dimensionless strain, their AIC and BIC values at these viscoelastic levels are also very close and directly comparable, which is fully consistent with the fact that the proposed model degenerates to a Burgers-type formulation below the long-term strength.

The most prominent advantage of the proposed model is apparent at the accelerating creep levels, i.e., the final loading level of each specimen where the axial stress exceeds the long-term strength. As shown in Table 5, the Burgers model completely fails to capture the accelerating creep: its R^2^ drops sharply to around 0.5, and the RMSE increases by more than one order of magnitude. This is because the Burgers model possesses neither a long-term strength threshold nor a nonlinear viscoplastic component and therefore can only represent linearly increasing steady-state creep rather than the upward-curving tertiary creep. In contrast, the proposed model retains a high fitting accuracy at these accelerating levels: its *R*^2^ remains between 0.836 and 0.999, and the *RMSE* is reduced by approximately 31–96% relative to the Burgers model, confirming that the introduced nonlinear viscoplastic component and the moisture–stress coupled damage mechanism are essential and effective for reproducing the accelerating creep stage. With the AIC and BIC recomputed on identical datasets using the unified dimensionless strain, the proposed model yields clearly more negative AIC and BIC values than the Burgers model at the accelerating levels. For instance, for specimen A-1 at the final level, the Burgers model gives AIC = −935.71 and BIC = −929.09, whereas the proposed model gives AIC = −1042.27 and BIC = −1029.04; for specimen A-3 at the final level, the corresponding values are −86.95 and −87.11 for the Burgers model versus −126.24 and −126.57 for the proposed model. The agreement among R^2^, RMSE, AIC, and BIC therefore confirms the genuine superiority of the proposed model in characterizing accelerating creep.

In summary, the proposed model not only preserves the Burgers model’s ability to describe viscoelastic creep below the long-term strength but also successfully overcomes its inherent limitation in characterizing accelerating creep, thereby verifying the rationality and advancement of the proposed nonlinear viscoelastic–plastic creep constitutive model.

## 6. Discussion

To place the present findings in a broader context, the creep behavior of the argillaceous siltstone investigated here is compared with recent experimental results reported for other red-bed rocks under coupled water-content and confining-pressure conditions, namely, the studies of Miao et al. [16] on sandstone and Xie et al. [46] on red-bed silty mudstone. The comparison focuses on the reduction in long-term strength, the steady-state creep rate, the water-induced changes in shear strength parameters, and the constitutive modeling approach.

Regarding long-term strength, the saturated specimens in this study exhibited a 28.4–37.5% reduction in long-term strength compared with the air-dried specimens under the same confining pressure, and the ratio of long-term strength to short-term peak strength ranged from 0.76 to 0.94. Based on creep tests on sandstone with water contents of 0–3.3% and confining pressures of 0–12 MPa, Miao et al. [16] reported that the critical strength entering the nonlinear acceleration stage—i.e., the long-term strength—was approximately 0.59–0.74 of the compressive strength, and that water content caused an exponential decrease in strength. Studying red-bed silty mudstone under multistage creep loading with AE monitoring, Xie et al. [46] found that the long-term strength remained within 64–75% of the compressive strength and was close to the crack damage stress. The long-term-strength ratios obtained in the present study (0.76–0.94) are therefore somewhat higher than those reported by Miao et al. [16] and Xie et al. [46]. Part of the difference can be attributed to lithological and methodological factors: the tested rock is an argillaceous siltstone, which is generally stronger and less water-sensitive than the red-bed silty mudstone investigated by Xie et al. [46]. More importantly, because both the long-term strength and the short-term strength were each determined from only one specimen per condition, strong discreteness is unavoidable. Therefore, the ratios of 0.76–0.94 need to be confirmed by replicate tests in future work. Nevertheless, the direction of the effect of water is consistent across these studies: water-wetting markedly reduces long-term strength, and increasing confining pressure improves it.

With respect to the steady-state creep rate, the axial strain rates measured in this study are on the order of 10^−6^/h, ranging from 0.6 to 2.9 × 10^−6^/h for air-dried specimens to 0.7–4.6 × 10^−6^/h for saturated specimens, and they increase with increasing axial stress. Xie et al. [46] reported that the steady-state creep rate of red-bed silty mudstone is highly sensitive to deviatoric stress and water content, which is in good agreement with the present observation that the steady-state rate increases with both axial (deviatoric) stress and water content. Miao et al. [16] similarly showed that creep deformation and the steady-state strain rate increase with increasing water content. The fact that the saturated specimens in this study consistently exhibit higher steady-state rates than the air-dried specimens therefore corroborates the water-enhanced creep deformation reported for sandstone and red-bed silty mudstone. In addition, the observation that increasing confining pressure suppresses the steady-state creep rate of the air-dried specimens is consistent with the confining-pressure-constrained creep behavior documented in the literature.

The present results further show that water-wetting reduces the long-term cohesion from 6.72 MPa (air-dried) to 2.66 MPa (saturated), a decrease of 60.4%, whereas the internal friction angle changes only slightly, from 42.25° to 41.15°. This indicates that the water-induced deterioration of long-term shear strength mainly results in a reduction in cohesion rather than altered frictional characteristics. Miao et al. [16] reported exactly analogous behavior for sandstone: water content significantly affected the micro-cohesion and internal friction angle, with cohesion being much more sensitive to water than the friction angle. The consistency between the two independent studies—on sandstone and on the argillaceous siltstone tested here—strongly supports the interpretation that water mainly weakens the intergranular cementation of the rock, while having a limited influence on the frictional resistance.

Finally, from the modeling viewpoint, the nonlinear viscoelastic–plastic creep model proposed in this study, which incorporates a moisture–stress coupled damage mechanism and a nonlinear viscoplastic component based on the Burgers model, is able to describe the whole creep process with R^2^ mostly greater than 0.90. Xie et al. [46] recently developed a unified nonlinear creep damage-coupled model by combining statistical damage theory with fractional calculus, and, unlike traditional models, their model parameters are stress-independent and can capture the coupling effects of water and confining pressure. In the present model, by contrast, the parameters are identified separately for each stress level and therefore vary with the stress level and the water-bearing state. Eliminating the stress dependence of the parameters—i.e., developing a model with stress-independent parameters as achieved by Xie et al. [46]—is an important direction for future improvement. Nevertheless, both the present model and that of Xie et al. [46] confirm that a damage-coupled framework is necessary and effective for reproducing the full-spectrum nonlinear creep of red-bed soft rocks under the combined action of water and confining pressure.

In summary, the triaxial creep results obtained for the red-bed argillaceous siltstone in this study are generally consistent with the literature on red-bed sandstones and mudstones: water-wetting reduces the long-term strength and increases the steady-state creep rate, and cohesion is much more sensitive to water than the internal friction angle. The slightly higher long-term-strength ratios reflect the specific lithology and the confining-pressure range. These comparisons place the present results within the existing body of knowledge and highlight both the commonality of water-induced creep deterioration in red-bed rocks and the specific characteristics of the argillaceous siltstone investigated here.

## 7. Conclusions

Based on step-loading triaxial compression creep tests of argillaceous siltstone from the dam site of a hydropower project in the lower Minjiang River, the creep deformation characteristics, long-term strength, and failure modes of argillaceous siltstone under different water-bearing states and confining pressures were systematically studied. Based on the Burgers model, a moisture–stress coupled time-dependent damage mechanism and a nonlinear viscoplastic component were introduced to construct a three-dimensional nonlinear viscoelastic–plastic creep constitutive model. The main conclusions are as follows:

(1) Water-wetting led to a marked deterioration in the creep mechanical properties of the argillaceous siltstone. Under the same confining pressure, the long-term strength of saturated specimens was reduced by 28.4% to 37.5% compared with air-dried specimens, and the axial strain rate in the steady-state creep stage was generally higher than that of air-dried specimens, suggesting that an increase in water content tends to weaken the long-term bearing capacity of argillaceous siltstone and accelerate its time-dependent deformation.

(2) Confining pressure exerted a notable constraining and enhancing effect on the creep characteristics of argillaceous siltstone. As the confining pressure increased from 4 MPa to 8 MPa, the creep failure axial stress of air-dried specimens increased from 56.02 MPa to 81.09 MPa, and the long-term strength increased from 52.50 MPa to 72.90 MPa. The instantaneous elastic modulus and viscoelastic parameters generally increased, indicating that, for the tested range, lateral restraint is beneficial for suppressing creep deformation and improving long-term stability.

(3) Under the tested conditions, the effect of water-wetting on the long-term shear strength was primarily characterized by a considerable reduction in cohesion, from 6.72 MPa in the air-dried state to 2.66 MPa in the saturated state, a decrease of 60.4%. In contrast, the internal friction angle changed only slightly, from 42.25° to 41.15°, suggesting that the reduction in long-term strength is possibly due to the degradation of cementing bonds rather than alteration of frictional characteristics. Further microstructural investigation would be required to verify this interpretation.

(4) The nonlinear viscoelastic–plastic creep constitutive model considering moisture–stress coupled time-dependent damage proposed in this study can reasonably describe the entire process of decelerating creep, steady-state creep, and accelerating creep of argillaceous siltstone under the deviatoric stress levels tested. The coefficients of determination *R*^2^ are mostly greater than 0.90, indicating a good descriptive capability of the model for the present dataset.

It should be noted that the conclusions are subject to several limitations. First, only one specimen was tested per combination of water-bearing state and confining pressure, so the observed trends may be affected by specimen-to-specimen variability and require confirmation using replicate tests. Second, the confining pressures were limited to 4, 6, and 8 MPa, so the derived long-term cohesion and internal friction angle are preliminary estimates restricted to this narrow range. Third, the constitutive model parameters were identified separately for each load level, so the model is, in practice, a group of fits with stress-dependent parameters. Developing a stress-independent formulation is an important task for future work. Finally, the model was calibrated and evaluated on the same dataset, and no independent validation on additional specimens or an external dataset was performed.

## Figures and Tables

**Table 1:** Basic specimen information.

Specimen No.	Water-Bearing State	Wave Velocity (m/s)	Mass (g)	Water Content (%)
A-1	Air-dried	1347	485.75	1.26
A-2	Air-dried	1590	478.67	1.23
A-3	Air-dried	1307	466.87	1.21
S-1	Saturated	1922	499.43	4.37
S-2	Saturated	1659	489.44	5.23
S-3	Saturated	1710	486.44	4.86

**Table 2:** Step-loading scheme for triaxial compression creep tests of argillaceous siltstone under different water-bearing states.

Specimen No.	Water-Bearing State	Confining Pressure *σ*_3_ (MPa)	Axial Stress of Step-Loading *σ*_1_ (MPa)					
			Level 1	Level 2	Level 3	Level 4	Level 5	Level 6
A-1	Air-dried	4	29.46	37.10	44.74	52.38	56.02	\
A-2	Air-dried	6	41.65	49.29	56.93	64.31	\	\
A-3	Air-dried	8	44.54	53.38	60.80	66.55	71.35	81.09
S-1	Saturated	4	16.73	21.83	26.92	32.01	37.10	\
S-2	Saturated	6	21.28	28.92	37.07	44.71	\	\
S-3	Saturated	8	28.37	38.56	46.20	51.29	56.38	\

**Table 3:** Summary of long-term strength parameters.

Lithology	Water-Bearing State	Long-Term Cohesion *c* (MPa)	Long-Term Internal Friction Angle *φ* (°)
Argillaceous siltstone	Air-dried	6.72	42.25
	Saturated	2.66	41.15

**Table 4:** Identified parameters of the three-dimensional creep constitutive model.

Specimen No.	*ω* /%	*σ*_3_ /MPa	*σ*_1_ − *σ*_3_ /MPa	*α* /(MPa·h)^−1^	*G*_1_ /GPa	*H*_1_ /GPa·h	*G*_2_ /GPa	*K*_2_ /GPa	*H*_2_ /GPa·h	*H*_3_ /GPa·h	*n* /h^−1^	R^2^
A-1	1.26	4.00	25.46	-	33.653	55.546	2.095	2.976	1.08 × 10^4^	-	-	0.969
			33.10	-	25.090	54.916	2.368	3.365	5.93 × 10^3^	-	-	0.952
			40.74	-	26.493	71.645	2.497	3.549	9.89 × 10^3^	-	-	0.962
			48.38	-	24.700	71.164	2.718	3.863	8.09 × 10^3^	-	-	0.968
			52.02	0.0188	21.196	39.663	2.598	3.692	5.71 × 10^3^	3.45 × 10^10^	2.012	0.921
A-2	1.23	6.00	35.65	-	39.704	44.369	2.663	3.785	1.37 × 10^4^	-	-	0.922
			43.29	-	28.507	46.696	2.881	4.095	8.61 × 10^3^	-	-	0.948
			50.93	-	22.889	75.837	2.996	4.258	6.62 × 10^3^	-	-	0.972
			58.31	0.069	35.242	52.989	3.065	4.355	6.30 × 10^3^	3.32 × 10^27^	16.717	0.998
A-3	1.21	8.00	36.54	-	331.607	1648.244	2.509	3.566	1.11 × 10^20^	-	-	0.942
			45.38	-	53.767	132.099	2.650	3.766	8.92 × 10^3^	-	-	0.839
			52.80	-	36.317	123.845	2.903	4.125	1.64 × 10^4^	-	-	0.912
			58.55	-	23.154	216.393	2.980	4.235	4.15 × 10^4^	-	-	0.913
			63.35	-	26.473	192.389	3.097	4.401	1.58 × 10^4^	-	-	0.848
			73.09	1.089	1210.302	92.503	3.161	4.492	1.23 × 10^1^	6.50 × 10^30^	552.561	0.999
S-1	4.37	4.00	12.73	-	19.276	46.154	1.347	3.608	3.42 × 10^3^	-	-	0.960
			17.83	-	14.721	45.644	1.603	4.294	5.04 × 10^3^	-	-	0.965
			22.92	-	12.445	45.860	1.845	4.943	3.55 × 10^3^	-	-	0.976
			28.01	-	10.117	44.409	2.083	5.578	3.05 × 10^3^	-	-	0.979
			33.10	0.0239	9.461	35.930	2.234	5.983	2.64 × 10^3^	2.38 × 10^9^	2.566	0.963
S-2	5.23	6.00	15.28	-	48.419	41.894	2.642	7.076	1.38 × 10^4^	-	-	0.927
			22.92	-	75.422	34.839	2.844	7.619	3.45 × 10^3^	-	-	0.975
			31.07	-	16.955	67.008	2.875	7.701	3.00 × 10^3^	-	-	0.966
			38.71	0.2601	243.240	20.509	2.928	7.842	2.64 × 10^3^	23.29	1.33 × 10^−7^	0.836
S-3	4.86	8.00	20.37	-	75.638	85.540	2.739	7.336	9.05 × 10^3^	-	-	0.978
			30.56	-	48.304	93.050	3.375	9.041	6.72 × 10^3^	-	-	0.983
			38.2	-	34.741	118.774	3.787	10.144	5.61 × 10^3^	-	-	0.991
			43.29	-	24.499	99.117	4.129	11.060	5.50 × 10^3^	-	-	0.990
			48.38	0.1644	403.818	61.307	4.321	11.574	7.73 × 10^20^	1.38 × 10^39^	18.008	0.911

**Table 5:** Comparison of fitting performance between the Burgers model and the proposed model.

Specimen No.	*ω* /%	*σ*_3_ /MPa	*σ*_1_ − *σ*_3_ /MPa	Duration /h	*N*	Burgers Model					Proposed Model				
						*k*	*R^2^*	*RMSE* /%	AIC	BIC	*k*	*R^2^*	*RMSE* /%	AIC	BIC
A-1	1.26	4.00	25.46	96	108	3	0.966	0.0015	−2393.21	−2385.17	3	0.969	0.0013	−2427.62	−2419.57
			33.10	96	109	3	0.964	0.0032	−2250.25	−2242.18	3	0.952	0.0037	−2217.96	−2209.89
			40.74	96	109	3	0.968	0.0033	−2243.54	−2235.47	3	0.962	0.0036	−2225.67	−2217.59
			48.38	96	109	3	0.972	0.0041	−2196.22	−2188.15	3	0.968	0.0043	−2184.66	−2176.59
			52.02	13	67	3	0.577	0.0887	−935.71	−929.09	6	0.921	0.0383	−1042.27	−1029.04
A-2	1.23	6.00	35.65	96	110	3	0.926	0.0022	−2353.38	−2345.28	3	0.922	0.0023	−2346.86	−2338.76
			43.29	96	110	3	0.959	0.0032	−2270.95	−2262.85	3	0.948	0.0036	−2242.68	−2234.58
			50.93	96	110	3	0.973	0.0048	−2181.75	−2173.65	3	0.972	0.0049	−2176.04	−2167.94
			58.31	4	21	3	0.508	0.1327	−272.24	−269.11	6	0.998	0.0082	−383.35	−377.08
A-3	1.21	8.00	36.54	144	91	3	0.952	0.0019	−1972.54	−1965.00	3	0.942	0.0031	−1885.89	−1878.36
			45.38	162	100	3	0.916	0.0046	−1991.37	−1983.56	3	0.839	0.0063	−1926.94	−1919.13
			52.80	144	91	3	0.923	0.0048	−1803.86	−1796.33	3	0.912	0.0051	−1792.33	−1784.80
			58.55	168	103	3	0.939	0.0073	−1956.16	−1948.26	3	0.913	0.0087	−1920.89	−1912.99
			63.35	168	103	3	0.909	0.0094	−1904.08	−1896.17	3	0.848	0.0122	−1850.77	−1842.86
			73.09	0.14	7	3	0.521	0.1308	−86.95	−87.11	6	0.999	0.0051	−126.24	−126.57
S-1	4.37	4.00	12.73	96	107	3	0.973	0.0026	−2253.29	−2245.27	3	0.960	0.0032	−2207.86	−2199.84
			17.83	96	107	3	0.971	0.0043	−2145.62	−2137.60	3	0.965	0.0047	−2127.15	−2119.14
			22.92	96	107	3	0.977	0.0062	−2067.31	−2059.29	3	0.976	0.0064	−2059.51	−2051.49
			28.01	96	107	3	0.979	0.0091	−1985.20	−1977.18	3	0.979	0.0092	−1983.75	−1975.73
			33.10	9	46	3	0.864	0.0651	−669.00	−663.52	6	0.963	0.0339	−722.99	−712.02
S-2	5.23	6.00	15.28	96	111	3	0.928	0.0016	−2445.53	−2437.40	3	0.927	0.0016	−2447.14	−2439.01
			22.92	96	111	3	0.977	0.0040	−2242.11	−2233.98	3	0.975	0.0041	−2234.04	−2225.91
			31.07	96	111	3	0.977	0.0099	−2040.93	−2032.80	3	0.966	0.0118	−2002.45	−1994.32
			38.71	2	13	3	0.658	0.2565	−149.11	−147.42	6	0.836	0.1777	−152.65	−149.26
S-3	4.86	8.00	20.37	96	110	3	0.982	0.0010	−2526.84	−2518.74	3	0.978	0.0011	−2499.15	−2491.05
			30.56	96	110	3	0.989	0.0019	−2385.64	−2377.53	3	0.983	0.0024	−2335.60	−2327.50
			38.2	96	109	3	0.991	0.0032	−2250.25	−2242.18	3	0.991	0.0031	−2259.11	−2251.04
			43.29	96	109	3	0.991	0.0049	−2157.36	−2149.29	3	0.990	0.0048	−2161.89	−2153.82
			48.38	5.2	28	3	0.543	0.0824	−391.68	−387.68	6	0.911	0.0362	−431.71	−423.72

## Data Availability

The original data presented in this study are included in the article. Further inquiries can be directed to the corresponding author.
